# ICAM-1 promotes T cell glycolytic reprogramming and tumor infiltration to drive ^18^F-FDG PET flares following radiotherapy

**DOI:** 10.1093/procel/pwaf111

**Published:** 2025-12-19

**Authors:** Rui Song, Meixin Zhao, Ting Zhang, Yining Zhang, Fuxin Guo, Huiying He, Haoyi Zhou, Kui Li, Jianze Wang, Jinhong Du, Feng Wang, Shixin Zhou, Hua Zhu, Jiadong Wang, Weifang Zhang, Zhi Yang, Zhaofei Liu

**Affiliations:** Department of Nuclear Medicine, Peking University Cancer Hospital and Department of Radiation Medicine, School of Basic Medical Sciences, Peking University, Beijing 100191, China; State Key Laboratory of Vascular Homeostasis and Remodeling, Department of Nuclear Medicine, Peking University Third Hospital, Beijing 100191, China; Department of Nuclear Medicine, Peking University Cancer Hospital and Department of Radiation Medicine, School of Basic Medical Sciences, Peking University, Beijing 100191, China; Department of Nuclear Medicine, Peking University Cancer Hospital and Department of Radiation Medicine, School of Basic Medical Sciences, Peking University, Beijing 100191, China; Department of Radiation Oncology, Peking University Third Hospital, Beijing 100191, China; Department of Pathology, Peking University Third Hospital, Beijing 100191, China; Department of Nuclear Medicine, Peking University Cancer Hospital and Department of Radiation Medicine, School of Basic Medical Sciences, Peking University, Beijing 100191, China; Department of Nuclear Medicine, Peking University Cancer Hospital and Department of Radiation Medicine, School of Basic Medical Sciences, Peking University, Beijing 100191, China; Department of Nuclear Medicine, Peking University Cancer Hospital and Department of Radiation Medicine, School of Basic Medical Sciences, Peking University, Beijing 100191, China; Department of Nuclear Medicine, Peking University Cancer Hospital and Department of Radiation Medicine, School of Basic Medical Sciences, Peking University, Beijing 100191, China; Department of Nuclear Medicine, Peking University Cancer Hospital and Department of Radiation Medicine, School of Basic Medical Sciences, Peking University, Beijing 100191, China; Key Laboratory of Carcinogenesis and Translational Research (Ministry of Education/Beijing), NMPA Key Laboratory for Research and Evaluation of Radiopharmaceuticals (National Medical Products Administration), Peking University Cancer Hospital, Beijing 100142, China; Department of Cell Biology, School of Basic Medical Sciences, Peking University, Beijing 100191, China; Department of Nuclear Medicine, Peking University Cancer Hospital and Department of Radiation Medicine, School of Basic Medical Sciences, Peking University, Beijing 100191, China; Key Laboratory of Carcinogenesis and Translational Research (Ministry of Education/Beijing), NMPA Key Laboratory for Research and Evaluation of Radiopharmaceuticals (National Medical Products Administration), Peking University Cancer Hospital, Beijing 100142, China; Department of Nuclear Medicine, Peking University Cancer Hospital and Department of Radiation Medicine, School of Basic Medical Sciences, Peking University, Beijing 100191, China; State Key Laboratory of Vascular Homeostasis and Remodeling, Department of Nuclear Medicine, Peking University Third Hospital, Beijing 100191, China; Department of Nuclear Medicine, Peking University Cancer Hospital and Department of Radiation Medicine, School of Basic Medical Sciences, Peking University, Beijing 100191, China; Key Laboratory of Carcinogenesis and Translational Research (Ministry of Education/Beijing), NMPA Key Laboratory for Research and Evaluation of Radiopharmaceuticals (National Medical Products Administration), Peking University Cancer Hospital, Beijing 100142, China; Department of Nuclear Medicine, Peking University Cancer Hospital and Department of Radiation Medicine, School of Basic Medical Sciences, Peking University, Beijing 100191, China; State Key Laboratory of Vascular Homeostasis and Remodeling, Department of Nuclear Medicine, Peking University Third Hospital, Beijing 100191, China; Key Laboratory of Carcinogenesis and Translational Research (Ministry of Education/Beijing), NMPA Key Laboratory for Research and Evaluation of Radiopharmaceuticals (National Medical Products Administration), Peking University Cancer Hospital, Beijing 100142, China

**Keywords:** positron emission tomography, ^18^F-fluorodeoxyglucose, radiotherapy, ICAM-1, flare phenomenon

## Abstract

^18^F-fluorodeoxyglucose (^18^F-FDG) is the most widely used radiotracer for positron emission tomography (PET) imaging in clinical oncology, owing to the elevated glycolytic activity of tumor cells. However, transient post-radiotherapy (RT) “metabolic flares” of ^18^F-FDG uptake are frequently observed in patients and are traditionally attributed to localized inflammatory responses. Whether these flares are linked to immune cell dynamics, particularly tumor-infiltrating T cells, and the mechanisms involved remain poorly understood. Here, we demonstrate that RT markedly upregulates intracellular adhesion molecule-1 (ICAM-1) expression and promotes T cell infiltration in tumors, as observed in both patients and mouse models. Genetic ablation of ICAM-1 significantly attenuates RT-induced metabolic flares in irradiated tumors, primarily due to reduced ^18^F-FDG uptake by tumor-infiltrating T cells rather than myeloid cells. Mechanistically, ICAM-1 engages with lymphocyte function-associated antigen 1 (LFA-1) to facilitate T cell clustering, thereby promoting their intratumoral accumulation and activating glycolysis and the tricarboxylic acid cycle via the PI3K-AKT-mTOR signaling pathway. These findings identify ICAM-1 as a critical regulator of T cell metabolic reprogramming and tumor infiltration following RT, offering a mechanistic explanation for ^18^F-FDG PET flares. Clinical monitoring of post-RT tumor ICAM-1 expression may enhance PET interpretation and aid in distinguishing pseudoprogression from true tumor progression.

## Introduction

Radiotherapy (RT) is one of the most widely used non-surgical cancer treatment modalities, applied in over half of all patients with cancer (Abdel-Wahab et al., 2024; Chandra et al., 2021). Its primary antitumor effect is mediated through DNA damage in tumor cells (Herrera et al., 2017). Beyond its cytotoxic properties, RT also exerts potent immunomodulatory effects by promoting the release of tumor-associated antigens and inducing cytokine secretion, thereby activating cytotoxic T cells and remodeling the tumor microenvironment (TME) (McLaughlin et al., 2020; Rodriguez-Ruiz et al., 2020).

Positron emission tomography (PET) using ^18^F-fluorodeoxyglucose (^18^F-FDG), a glucose analog, is the most commonly employed molecular imaging tool for noninvasive visualization of primary tumors, residual disease, and metastases, as well as for tumor staging and monitoring responses to therapies, including RT (Bai et al., 2023; Bussink et al., 2011). ^18^F-FDG PET leverages the “Warburg effect” (Vander Heiden and Deberardinis, 2017), characterized by elevated aerobic glycolysis in tumor cells, for tumor detection. Because metabolic alterations often precede anatomical changes, ^18^F-FDG PET offers superior sensitivity compared to anatomical imaging modalities such as computed tomography (CT) and magnetic resonance imaging (MRI), particularly for early assessment of treatment response (Basu and Alavi, 2007; Weber, 2009). However, ^18^F-FDG PET lacks tumor specificity, and elevated uptake may also occur in inflammatory or infectious conditions while signal intensity may be reduced in tumors with low metabolic activity (Ben-Haim and Ell, 2009; Pijl et al., 2021). Notably, a well-recognized phenomenon termed “metabolic flare,” involving transient increases in ^18^F-FDG uptake in tumors responding to RT, is frequently observed in clinical practice (Ben-Haim and Ell, 2009; Haberkorn et al., 1991). For instance, a prospective study of patients with locally advanced non-small cell lung cancer reported metabolic flare in 41% of cases (van Baardwijk et al., 2007). These flares, which do not reflect tumor progression, have traditionally been attributed to RT-induced local inflammation and may persist for weeks to months, thereby complicating image interpretation (Haberkorn et al., 1991; Rahman et al., 2019).

In addition to recruiting inflammatory myeloid cells such as neutrophils, activated macrophages, and myeloid-derived suppressor cells (MDSCs), RT can also promote the infiltration of adaptive immune cells, particularly T cells, into both irradiated and distant non-irradiated lesions. This immune infiltration underlies the development of systemic antitumor immunity and the abscopal effect of RT (Herrera et al., 2017; Rodríguez-Ruiz et al., 2018). Intriguingly, emerging evidence indicates that immune cells within the TME can exhibit higher glucose uptake than tumor cells (Reinfeld et al., 2021), raising the possibility that immune infiltration, rather than inflammation alone, may contribute to ^18^F-FDG flares following RT.

Our recent work revealed that RT induces CD8^+^ T cell infiltration and upregulates intracellular adhesion molecule-1 (ICAM-1) expression in both irradiated and distant tumors (Zhao et al., 2021), implicating ICAM-1 as a mediator of RT-induced immune responses. ICAM-1 is known to be expressed on dendritic cells (DCs), myeloid cells, and endothelial cells, where it facilitates immune cell migration through interaction with lymphocyte function-associated antigen-1 (LFA-1) (Dustin, 2019; Ramos et al., 2014). However, the specific role of ICAM-1 expressed by T cells in the TME following RT remains poorly understood (Zhang et al., 2024; Zhao et al., 2021). In this study, we examined whether T cell infiltration contributes to ^18^F-FDG PET flares and investigated the role of T cell-intrinsic ICAM-1 in regulating T cell metabolic activity. We found that ICAM-1 on T cells promotes ^18^F-FDG accumulation by facilitating T cell clustering through ICAM-1 and LFA-1 interaction, enhancing tumor infiltration, and driving glycolytic reprogramming. These findings identify ICAM-1 as a potential metabolic marker of activated T cells and support the development of ICAM-1-targeted imaging approaches to complement ^18^F-FDG PET, thereby improving the interpretation of PET signals and minimizing the risk of misdiagnosing pseudoprogression in tumors after RT.

## Results

### RT induces ^18^F-FDG flares and upregulates ICAM-1 expression in tumors of patients

**Figure 1.**
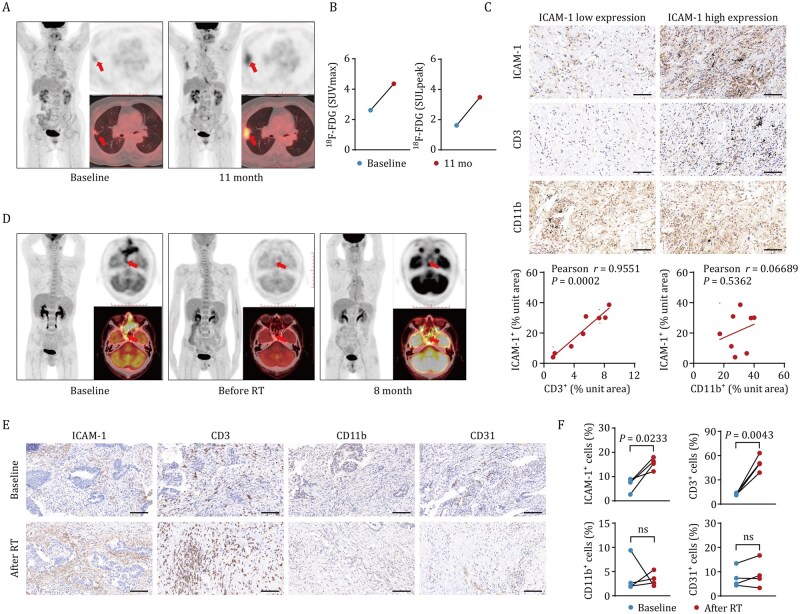
**RT induces metabolic flares of ^18^F-FDG and upregulates ICAM-1 expression in tumors of patients**. (A) ^18^F-FDG PET/CT images of a 70-year-old woman (patient #1; Table S1) with lung cancer (clinical stage cT1N0M0) receiving stereotactic RT (95% PTV, 50 Gy in 5 fractions). At baseline, the nodule showed an SUV_max_ of 2.61 and an SUL_peak_ of 1.62. Eleven months after RT, PET/CT revealed increased uptake (SUV_max_: 4.36; SUL_peak_: 3.48), indicative of PMD per PERCIST criteria. Tumors are indicated by red arrows. (B) Changes in SUV_max_ and SUL_peak_ values of ^18^F-FDG PET before and after RT in patient #1. (C) Immunohistochemical staining of ICAM-1, CD3, and CD11b in ICAM-1 low- and high-expressing regions of the post-RT tumor from patient #1. Correlation between ICAM-1 and CD3 (*r *= 0.9551) and correlation between ICAM-1 and CD11b (*r *= 0.06689) were determined by Pearson’s correlation analysis (*n *= 8). Scale = 100 μm. (D) ^18^F-FDG PET/CT images of a 55-year-old man (patient #2; Table S1) with NK/T-cell lymphoma (stage IVB). At baseline, SUV_max_ and SUL_peak_ of the nasopharyngeal lesions were 8.90 and 5.92, respectively. Midterm ^18^F-FDG PET/CT revealed nearly complete response after chemotherapy, while post-RT ^18^F-FDG PET/CT (50 Gy in 25 fractions) showed new hypermetabolic lesions (SUV_max_: 4.12; SUL_peak_: 4.37). Tumors are indicated by red arrows. (E and F) Representative immunohistochemical staining (E) and quantification (F) of ICAM-1, CD3, CD11b, and CD31 in tumor tissues (Table S2) before and after RT (*n *= 4 per group). Scale = 100 μm. *P* values were determined by a paired Student’s *t* test (F). ns, not significant (*P *> 0.05).

^18^F-FDG uptake reflects cellular glucose metabolism and is widely used in PET imaging to monitor tumor progression. However, its ability to distinguish proliferating tumor cells from inflammatory infiltrates remains limited (Ben-Haim and Ell, 2009; Rahman et al., 2019). We retrospectively reviewed PET images from patients who had received RT at our institution and selected two representative cases (Table S1). In the first case (Fig. 1A), a 70-year-old woman with stage cT1N0M0 lung cancer presented a 1.77 × 1.46 cm nodule in the right lung. Baseline ^18^F-FDG PET revealed a maximum standardized uptake value (SUV_max_) of 2.61 and a peak SUV corrected for lean body mass (SUL_peak_) of 1.62. Eleven months after stereotactic RT (50 Gy in 5 fractions), ^18^F-FDG PET showed markedly increased SUV_max_ (4.36) and SUL_peak_ (3.48) in the same lesion (Fig. 1B), suggesting progressive metabolic disease (PMD) per the PET Response Criteria in Solid Tumors (PERCIST) (Wahl et al., 2009). However, biopsy revealed no residual tumor, and immunohistochemistry showed that ICAM-1 expression closely associated with CD3 (a T cell marker). Quantitative analysis confirmed a strong correlation between ICAM-1 and CD3 (Pearson’s *r *= 0.9551; Fig. 1C), but not with CD11b (a myeloid cell marker; Pearson’s *r *= 0.06689; Fig. 1C).

In the second case (Fig. 1D), a 55-year-old man with stage IVB NK/T-cell lymphoma received chemotherapy followed by RT. Baseline ^18^F-FDG PET showed an SUV_max_ of 8.90 and an SUL_peak_ of 5.92 in the nasopharynx and paranasal sinuses. After two cycles of chemotherapy, interim ^18^F-FDG PET showed near-complete lesion resolution. Following RT (50 Gy in 25 fractions) and two additional chemotherapy cycles, ^18^F-FDG PET/CT at 8 months revealed elevated ^18^F-FDG uptake (SUV_max_ 4.12; SUL_peak_ 4.37) in the same region. Although this was categorized as progressive disease (PD) per Lugano criteria (Van Heertum et al., 2017), nasal endoscopic biopsy revealed inflammatory infiltrates without residual lymphoma. Consistent with the first case, immunohistochemistry demonstrated that ICAM-1 expression was associated with CD3-positive T cells but not with CD11b-positive myeloid cells (Fig. S1). Together, these results suggest that RT-induced immune infiltration can drive transient ^18^F-FDG flares independent of actual tumor progression, highlighting the need for caution when interpreting PERCIST- and Lugano-based response assessments after RT.

To further investigate ICAM-1 dynamics after RT, we analyzed tumor specimens from four rectal adenocarcinoma patients before and after RT (Table S2). Immunohistochemistry revealed significantly increased ICAM-1^+^ cells in post-RT tumors. This upregulation coincided with increased CD3^+^ T cells, but not CD11b^+^ myeloid cells or CD31^+^ endothelial cells (Fig. 1E and 1F), supporting that RT promotes ICAM-1 expression and T cell infiltration.

### RT induces upregulation of ICAM-1 predominantly on T cells

Building on clinical findings, we next evaluated ICAM-1 expression profiles in tumors and systemically in mouse models using whole-body PET imaging. To this end, we synthesized an ICAM-1-specific radiotracer ^89^Zr-DFO-αICAM-1/Fab by conjugating the Fab fragment of an anti-ICAM-1 antibody with deferoxamine (DFO) followed by radiolabeling with ^89^Zr (Fig. S2). ^89^Zr-DFO-αICAM-1/Fab (specific activity: ∼1.48 MBq/μg) exhibited high radiochemical purity (>98%) and excellent *in vitro* stability for up to 48 h (Fig. S3A and S3B). Specificity for ICAM-1 of ^89^Zr-DFO-αICAM-1/Fab was confirmed by *in vitro* binding assays (Fig. S3C) and *in vivo* PET imaging, which showed significantly higher tumor uptake in MC38 tumor-bearing mice compared with an isotype control ^89^Zr-DFO-IgG/Fab (Fig. S4A and S4B).

**Figure 2.**
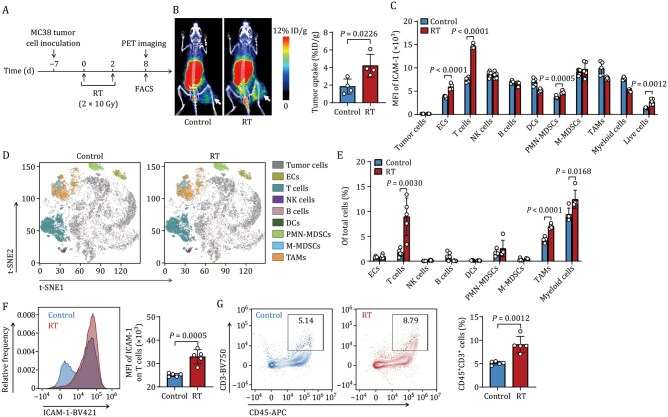
**RT induces upregulation of ICAM-1 predominantly on T cells**. (A) Experimental schedule for RT, *in vivo* PET imaging using ^89^Zr-DFO-αICAM-1/Fab, and flow cytometry in MC38 tumor-bearing mice. (B) Representative PET/CT images and quantitative analysis of tumor uptake of ^89^Zr-DFO-αICAM-1/Fab at 24 h post-injection (*n *= 4 per group). Tumors are indicated by white arrows. (C–E) Flow cytometric analysis of ICAM-1 expression on different cell subsets (C), t-SNE visualization (D), and quantification of cell population frequencies (E) in MC38 tumors (*n *= 5 per group). (F and G) Flow cytometric analysis of ICAM-1 expression on T cells (F) and frequencies of CD45^+^CD3^+^ T cells (G) in Lewis lung carcinomas (*n *= 5 per group). All the numerical data are presented as mean ± SD. *P* values were determined by an unpaired Student’s *t* test (B, C, and E–G). ECs, endothelial cells; NK cells, natural killer cells; PMN-MDSCs, polymorpho-nuclear myeloid-derived suppressor cells; M-MDSCs, monocytic myeloid-derived suppressor cells; TAMs, tumor associated macrophages.

Using this ICAM-1-specific radiotracer, PET imaging revealed significantly increased tumor uptake of ^89^Zr-DFO-αICAM-1/Fab in mice 8 days after two doses of 10 Gy RT (Fig. 2A and 2B), indicating enhanced ICAM-1 expression in tumors post-RT. *Ex vivo* immunofluorescence staining corroborated this finding, showing upregulated ICAM-1 and increased CD3^+^ T cells in tumor tissues after RT (Fig. S5A and S5B). Flow cytometry further revealed that ICAM-1 expression was upregulated on T cells (CD45^+^CD3^+^), endothelial cells (CD31^+^), and polymorpho-nuclear MDSCs (PMN-MDSCs; CD45^+^CD11b^+^Ly6G^+^Ly6C^-^) rather than other cell types on day 8 after RT (Fig. 2C).

Additionally, RT significantly increased the infiltration of T cells (CD45^+^CD3^+^), total myeloid cells (CD45^+^CD11b^+^), and tumor associated macrophages (TAMs; CD45^+^CD11b^+^F4/80^+^) (Fig. 2D and 2E). Among these, T cells showed the highest increase in both number (Fig. 2E) and ICAM-1 expression (Fig. 2C) after RT. These results were validated in a separate Lewis lung carcinoma model, where RT led to marked upregulation of ICAM-1 on T cells (Fig. 2F) and increased T cell (CD45^+^CD3^+^) accumulation (Fig. 2G). Further flow cytometry analysis of T-cell subsets revealed that RT significantly upregulated ICAM-1 expression across all subsets (Fig. S6A), while predominantly increasing the infiltration of CD4^+^ (CD45^+^CD3^+^CD4^+^) and CD8^+^ T cells (CD45^+^CD3^+^CD8^+^) in MC38 tumors (Fig. S6B). These findings suggest that ICAM-1 upregulation after RT occurs predominantly in tumor-infiltrating T cells.

### ICAM-1 deficiency abrogates RT-induced ^18^F-FDG flares and reduces T cell-specific ^18^F-FDG uptake

**Figure 3.**
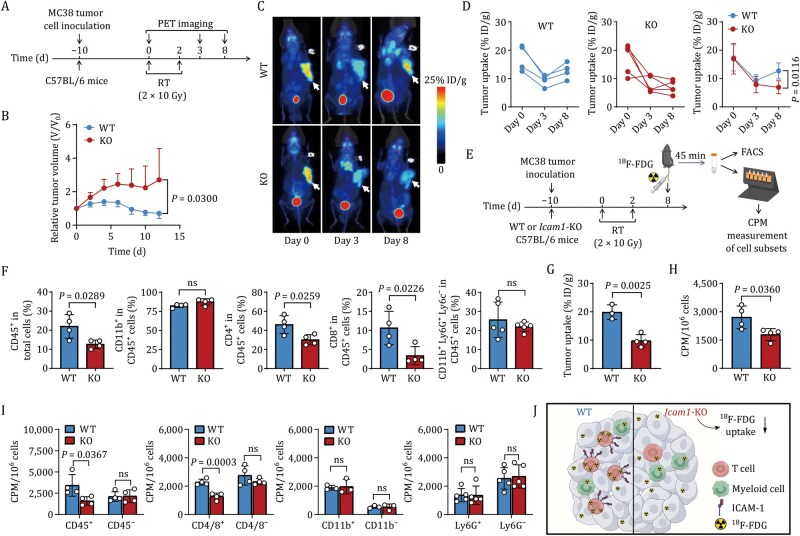
**Genetic ablation of ICAM-1 abrogates RT-induced ^18^F-FDG flares and reduces T cell glucose uptake**. (A) Experimental design of RT and PET imaging in WT or *Icam1*-KO C57BL/6 mice bearing MC38 tumors. (B) Tumor growth curves post-RT in WT and *Icam1*-KO mice (*n *= 4–5 per group). (C and D) Representative PET/CT images (C) and quantification of ^18^F-FDG tumor uptake (D) on days 0, 3, and 8 after RT in MC38 tumor-bearing WT or *Icam1*-KO C57BL/6 mice (*n *= 4–5 per group). Tumors are indicated by white arrows. (E) Workflow for RT, tumor tissue dissociation, flow cytometry, and cell sorting for measuring ^18^F-FDG avidity. (F) Flow cytometric analysis of immune cell subsets, including CD45^+^, CD11b^+^CD45^+^, CD4^+^CD45^+^, CD8^+^CD45^+^, and CD11b^+^Ly6G^+^Ly6C^-^CD45^+^ cells (*n *= 4–5 per group). (G–I) *Ex vivo*  ^18^F-FDG uptake in whole tumors (G), total cells dissociated from tumors (H), and sorted immune cell subsets (I) from WT and *Icam1*-KO mice on day 8 (*n *= 3–5 per group). (J) Schematic illustrating how ICAM-1 deletion impairs T cell glucose uptake and tumor ^18^F-FDG accumulation. All numerical data are presented as mean ± SD. *P* values were determined by two-way ANOVA (B) and an unpaired Student’s *t* test (D and F–I). ns, not significant (*P *> 0.05).

To determine whether ICAM-1 contributes to RT-induced ^18^F-FDG flares, we performed longitudinal ^18^F-FDG PET imaging on days 0, 3, and 8 post-RT in MC38 tumor-bearing wild type (WT) and *Icam1*-knockout (KO) C57BL/6 mice that received two doses of RT (Fig. 3A). Compared with WT mice, ICAM-1 deficiency impaired the antitumor effects of RT (Fig. 3B). While ^18^F-FDG uptake was comparable on days 0 and 3, WT mice showed significantly higher tumor uptake than *Icam1*-KO mice on day 8 (Fig. 3C and 3D). These results were replicated in Lewis lung carcinoma-bearing mice (Fig. S7A–D), confirming that ICAM-1 is essential for the post-RT ^18^F-FDG flare.

To determine the cellular contributors to ^18^F-FDG uptake, we analyzed tumor-infiltrating immune cells by flow cytometry and measured ^18^F-FDG uptake by magnetic-activated cell sorting on day 8 post-RT (Fig. 3E). *Icam1*-KO mice had reduced tumor infiltration of CD45^+^ cells and CD4^+^ and CD8^+^ T cells compared to WT, whereas myeloid cell, PMN-MDSC, DC, and macrophage populations remained unchanged (Figs. 3F, S8A–D, S9A and S9B). Consistent with PET findings, both *ex vivo* necropsy-based biodistribution analysis (Fig. 3G) and per-cell quantification of *in vivo*  ^18^F-FDG radioactivity (Fig. 3H) demonstrated significantly reduced uptake in tumors of *Icam1*-KO mice. Cellular analysis further revealed that ICAM-1 deficiency decreased ^18^F-FDG uptake in CD45^+^ and CD4/8^+^ T (both CD3^+^CD4^+^ and CD3^+^CD8^+^ populations) cell subsets, but not in CD45^-^ tumor cells, CD11b^+^ myeloid cell, Ly6G^+^ cell, CD11c^+^ DC, and F4/80^+^ macrophage subsets (Figs. 3I, S9C and S9D). Together, these results demonstrate that ICAM-1 expressed on T cells contributes to the ^18^F-FDG uptake in the tumors after RT (Fig. 3J).

### ICAM-1 inhibition abrogates ^18^F-FDG uptake in tumor-infiltrating T cells originated from lymphoid tissues

**Figure 4.**
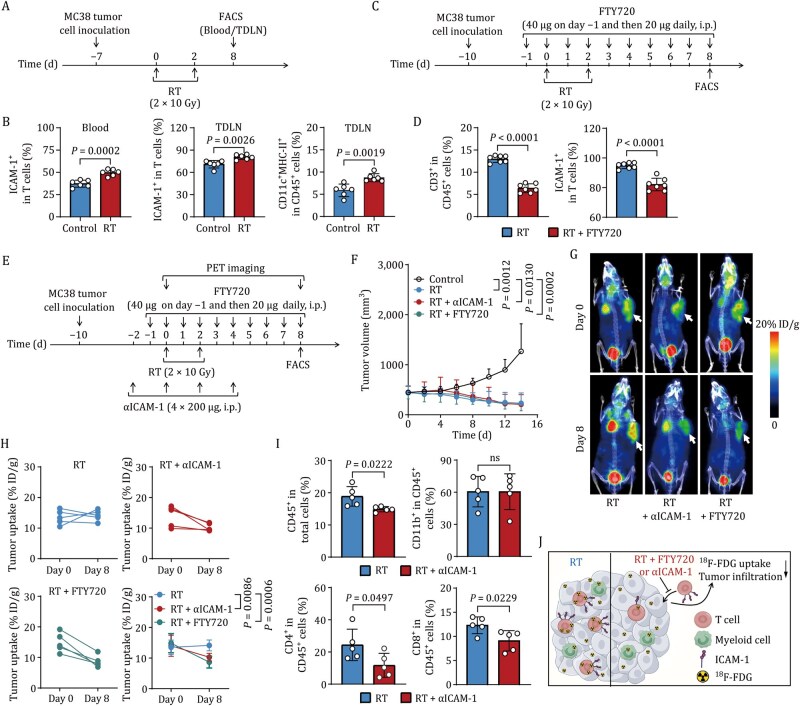
**Pharmacological ICAM-1 blockade abrogates ^18^F-FDG uptake in tumor-infiltrating T cells originated from lymphoid ­tissues**. (A) Experimental schedule for RT and flow cytometry of peripheral blood and tumor-draining lymph nodes (TDLNs) in MC38 tumor-bearing mice. (B) Flow cytometric analyses of ICAM-1^+^ T cells (ICAM-1^+^CD45^+^CD3^+^) in blood and TDLNs and activated dendritic cells (CD11c^+^MHC-II^+^CD45^+^) in TDLNs (*n *= 6 per group). (C) Experimental schedule of RT combined with FTY720 treatment. (D) Flow cytometry for T cells (CD3^+^CD45^+^) and ICAM-1^+^ T cells (ICAM-1^+^CD45^+^CD3^+^) in MC38 tumors (*n *= 7 per group). (E) Experimental schedule for RT combined with anti-ICAM-1 antibody and FTY720. (F) Tumor growth curves under indicated treatments: PBS (control), RT, RT + αICAM-1, and RT + FTY720 (*n *= 5–7 per group). (G and H) Representative PET/CT images (G) and tumor uptake quantification (H) of ^18^F-FDG on days 0 and 8 post-treatment (*n *= 5 per group). Tumors are indicated by white arrows. (I) Flow cytometric analysis of CD45^+^, CD11b^+^CD45^+^, CD4^+^CD45^+^, and CD8^+^CD45^+^ cells on day 8 (*n *= 5 per group). (J) Schematic showing ICAM-1 inhibition impairs T cell tumor infiltration and reduces ^18^F-FDG tumor uptake. All numerical data are presented as mean ± SD. *P* values were determined by an unpaired Student’s *t* test (B, D, and I), two-way ANOVA (F), and one-way ANOVA with a *post hoc* Tukey’s test (H). ns, not significant (*P *> 0.05).

We next investigated the origin of the ICAM-1^+^ T cells infiltrating tumors after RT. As RT induces immunogenic cell death that activates DCs and promotes T cell recruitment into the TME (Galluzzi et al., 2023), we hypothesized that the increased ICAM-1^+^ T cells were generated during RT-induced immune activation and subsequently migrated into tumors. To test this, we collected blood and tumor-draining lymph nodes (TDLNs) from MC38 tumor-bearing mice treated with or without RT and assessed ICAM-1^+^ T cells by flow cytometry (Fig. 4A). Compared to control, RT-treated mice showed a marked increase in ICAM-1^+^ T cells in both the blood and TDLNs, which correlated with increased activation of DCs in TDLNs (Figs. 4B, S10A and S10B). Moreover, *in vitro* co-culture of irradiated tumor lysates with bone marrow-derived DCs (BMDCs) and T cells significantly upregulated ICAM-1 expression on T cells relative to control lysates (Fig. S11A and S11B).

To determine whether these ICAM-1^+^ T cells were recruited from lymphoid tissues, we administered FTY720, a sphingosine-1-phosphate receptor (S1PR1) antagonist that blocks T cell egress from lymphoid organs (Ito et al., 2019; Zhou et al., 2020) (Fig. 4C). FTY720 treatment significantly reduced both total T cells and ICAM-1^+^ T cells in tumors, as shown by flow cytometry (Figs. 4D, S12A and S12B). Transwell assays further confirmed that ICAM-1 deficiency impaired T cell migration (Fig. S13A and S13B). These findings suggest that RT induces ICAM-1 upregulation on T cells, promoting their migration from lymphoid tissues into tumors.

To evaluate whether ICAM-1-mediated T cell infiltration contributes to ^18^F-FDG PET flares, we blocked ICAM-1 using a neutralizing antibody or inhibited T cell trafficking with FTY720 in RT-treated mice (Fig. 4E). Neither intervention compromised the antitumor efficacy of RT (Fig. 4F). However, both ICAM-1 blockade and FTY720 treatment significantly reduced ^18^F-FDG tumor uptake on day 8 post-RT, as shown by *in vivo* PET imaging (Fig. 4G and 4H). *Ex vivo* radioactivity measurements confirmed that the reduction in ^18^F-FDG uptake was confined to CD45^+^ immune cells, but not CD45^-^ tumor cells (Fig. S14). Flow cytometric analysis further revealed that blocking ICAM-1 markedly reduced tumor-infiltrating CD45^+^ cells and CD4^+^ and CD8^+^ T cells, but not CD11b^+^ myeloid cells (Figs. 4I and S15A–D). Near-infrared imaging and immunofluorescence staining confirmed that ICAM-1 blockade impaired the tumor infiltration of DiR- and CFSE-labeled T cells (Fig. S16A and S16B). Collectively, these results demonstrate that ICAM-1 upregulation following RT facilitates T cell infiltration and contributes to the ^18^F-FDG flares, which can be attenuated by blocking ICAM-1 or lymphoid egress (Fig. 4J).

### ICAM-1–LFA-1 interaction promotes T cell clustering and enhances tumor infiltration and ^18^F-FDG uptake

Having established that genetic and pharmacological ICAM-1 inhibition suppresses ^18^F-FDG tumor uptake, we next explored the underlying mechanisms. Since ICAM-1 is also expressed on host endothelial cells and facilitates the adhesion and transmigration of leukocytes via interaction with LFA-1, it is possible that ICAM-1 blockade interferes with the interactions between ICAM-1 on endothelial cells and LFA-1 on T cells (Sabatos et al., 2008), thereby impairing T cell infiltration. To exclude this possibility and investigate the role of ICAM-1 on T cells per se, we isolated OT-I T cells from CD45.2^+^ WT or *Icam1*-KO OT-I mice. Flow cytometric analysis confirmed ICAM-1 deficiency in *Icam1*-KO OT-I T cells (Fig. S17A). These cells were then adoptively transferred into MC38-ovalbumin (MC38-OVA) tumor-bearing CD45.1^+^ mice to assess the T cell-intrinsic role of ICAM-1 (Fig. S17B). Compared with control mice, adoptive transfer of WT OT-I T cells significantly suppressed tumor growth. In contrast, the antitumor effect was abrogated in mice receiving *Icam1*-KO OT-I T cells (Fig. S17C). In line with this, PET imaging on day 4 revealed markedly reduced tumor uptake of ^18^F-FDG in mice receiving *Icam1*-KO OT-I T cells compared to those receiving WT cells (Fig. S17D and S17E).

To directly compare the infiltration capacity of WT versus *Icam1*-KO T cells in the same host environment, we mixed CD45.2^+^ WT and *Icam1*-KO OT-I T cells at a 1:1 ratio and transferred them into MC38-OVA tumor-bearing CD45.1^+^ mice (Fig. S17F). Flow cytometric analysis on day 4 showed that the majority of tumor-infiltrating CD45.2^+^CD8^+^ T cells were ICAM-1-positive WT cells (96.1% ± 2.69%) rather than ICAM-1-deficient KO cells (3.51% ± 2.59%) (Fig. S17G) indicating that ICAM-1 deficiency directly impairs T cell tumor infiltration and reduces ^18^F-FDG tumor uptake.

**Figure 5.**
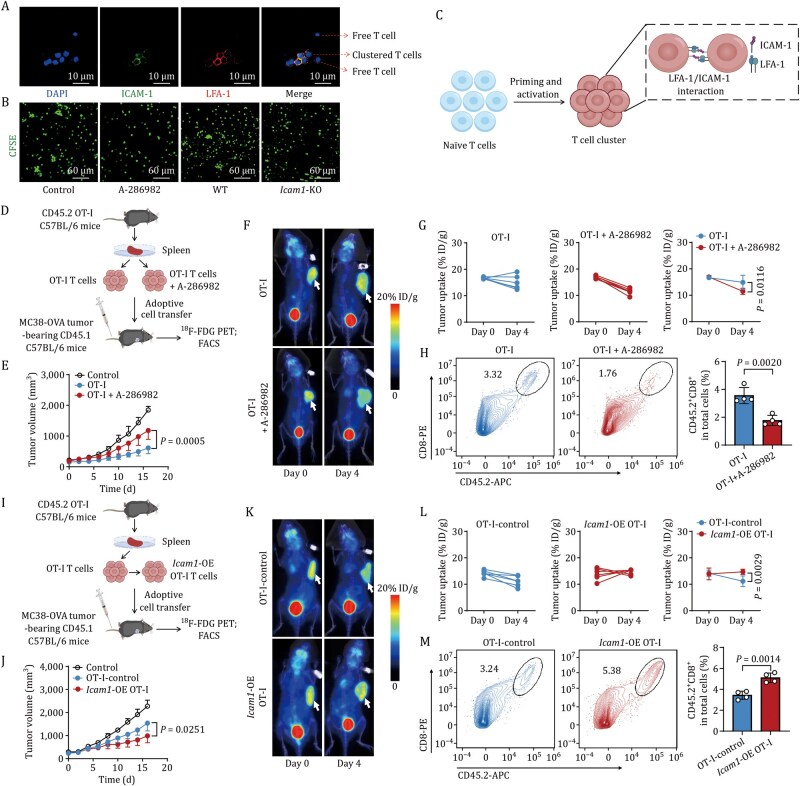
**ICAM-1–LFA-1 interaction promotes T cell clustering and tumor infiltration, and enhances ^18^F-FDG tumor uptake**. (A) ­Immunofluorescence staining of ICAM-1 and LFA-1 in clustered and free T cells. (B) ICAM-1–LFA-1-mediated clustering assessed in T cells cultured with or without A-286982 and in WT versus *Icam1*-KO T cells labeled with CFSE. (C) Schematic showing T cell clustering via ICAM-1–LFA-1 interactions. (D) Experimental design of adoptive transfer of CD45.2^+^ OT-I T cells (with or without A-286982 pre-treatment) into MC38-OVA tumor-bearing CD45.1^+^ C57BL/6 mice. (E) Tumor growth curves after treatment with PBS (control), OT-I T cells (OT-I), or OT-I T cells pre-treated with A-286982 (OT-I + A-286982) (*n *= 5–6 per group). (F and G) Representative PET/CT images (F) and ^18^F-FDG tumor uptake quantification (G) on days 0 and 4 post-transfer (*n* = 6 per group). (H) Representative flow cytometric plots and quantification of CD45.2^+^CD8^+^ T cells in tumors treated with OT-I or OT-I + A-286982 (*n *= 4 per group). (I) Experimental design for adoptive transfer of CD45.2^+^ OT-I T cells or *Icam1*-OE CD45.2^+^ OT-I T cells into MC38-OVA tumor-bearing CD45.1^+^ mice. (J) Tumor growth curves of MC38-OVA tumor-bearing mice following the indicated treatments: PBS (control), adoptive transfer of OT-I T cells transfected with control retrovirus (OT-I-control), and adoptive transfer of *Icam1*-OE CD45.2^+^ OT-I T cells (*Icam1*-OE OT-I) (*n *= 6 per group). (K and L) Representative PET/CT images (K) and ^18^F-FDG tumor uptake quantification (L) on days 0 and 4 (*n *= 6 per group). (M) Representative flow cytometric plots and quantification of CD45.2^+^CD8^+^ T cells in tumors treated with OT-I-control and *Icam1*-OE OT-I (*n *= 4 per group). Tumors are indicated by white arrows in the PET/CT images. All numerical data are presented as mean ± SD. *P* values were determined by two-way ANOVA (E and J) and an unpaired Student’s *t* test (G, H, L, and M).

While these findings highlight the contribution of ICAM-1 on T cells to tumor infiltration, T cells themselves express LFA-1, the binding partner of ICAM-1 (Sabatos et al., 2008), raising the possibility that ICAM-1 may also engage in homotypic interactions with LFA-1 on adjacent T cells. We thus hypothesized that ICAM-1–LFA-1 interactions on T cells may mediate T cell clustering, contributing to infiltration and metabolic activity. To test this, T cells were cultured in the presence or absence of A-286982, a selective inhibitor of ICAM-1–LFA-1 interaction (Wei et al., 2023). Immunofluorescence staining revealed that ICAM-1 expression and ICAM-1–LFA-1 co-localization were enhanced in clustered T cells compared to individual T cells (Fig. 5A). Treatment with A-286982 or genetic ICAM-1 deficiency significantly reduced T cell cluster formation, as visualized using CFSE-labeled T cells (Fig. 5B). These findings suggest that ICAM-1 expression promotes LFA-1-mediated T cell clustering during activation (Fig. 5C), which may contribute to tumor infiltration.

To assess the functional consequences of disrupting this interaction *in vivo*, CD45.2^+^ OT-I T cells pre-treated with A-286982 or vehicle were adoptively transferred into MC38-OVA tumor-bearing CD45.1^+^ mice (Fig. 5D). Disruption of the ICAM-1–LFA-1 interaction significantly impaired the antitumor activity of transferred T cells (Fig. 5E), reduced tumor uptake of ^18^F-FDG (Fig. 5F and 5G), and decreased CD45.2^+^ T cell infiltration as assessed by flow cytometry (Fig. 5H).

To determine whether ICAM-1 overexpression could enhance T cell clustering, infiltration, and metabolic activity, we transduced OT-I T cells with a retroviral vector encoding ICAM-1 (*Icam1*-OE) (Fig. S18A and S18B). *Icam1*-OE T cells formed more clusters than control vector-transduced cells (Fig. S19). In MC38-OVA tumor-bearing CD45.1^+^ mice, adoptive transfer of *Icam1*-OE OT-I T cells (Fig. 5I) significantly inhibited tumor growth (Fig. 5J) and was associated with higher ^18^F-FDG tumor uptake on day 4, as revealed by PET imaging (Fig. 5K and 5L). Flow cytometric analysis demonstrated markedly enhanced infiltration of CD45.2^+^CD8^+^ T cells in tumors of mice receiving *Icam1*-OE OT-I T cells compared with those receiving control OT-I T cells (Fig. 5M).

Collectively, these results demonstrate that ICAM-1–LFA-1 interactions between T cells promote T cell clustering, thereby enhancing tumor infiltration and increasing ^18^F-FDG tumor uptake. These findings suggest that RT-induced ICAM-1 upregulation may drive T cell accumulation and glucose metabolic activation within tumors.

### ICAM-1 reprograms glycolysis and enhances T cell effector function through the PI3K-AKT-mTOR signaling pathway

**Figure 6.**
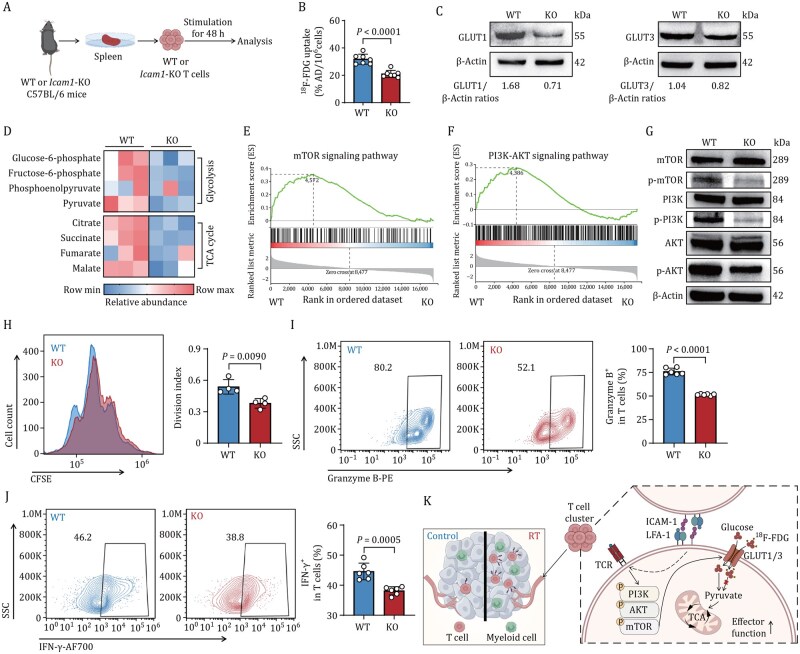
**ICAM-1 reprograms T cell glycolysis and enhances effector function via the PI3K-AKT-mTOR signaling pathway**. (A) ­Schematic of WT and *Icam1*-KO T cell isolation and preparation. (B) *In vitro*  ^18^F-FDG uptake in WT and *Icam1*-KO T cells (*n *= 7 per group). (C) Western blot analysis of GLUT1 and GLUT3 expression in WT and *Icam1*-KO T cells. (D) Targeted metabolomic analysis of glycolytic and TCA cycle intermediates in WT and *Icam1*-KO T cells (*n *= 3 per group). (E and F) GSEA of mTOR (E) and PI3K-AKT (F) ­signaling pathways in WT versus *Icam1*-KO T cells. (G) Western blot analysis of non-phosphorylated and phosphorylated mTOR, PI3K, and AKT in WT and *Icam1*-KO T cells. (H) Flow cytometric analysis of cell proliferation using CFSE dilution assay in WT and *Icam1*-KO T cells (*n *= 4 per group). (I and J) Flow cytometric analysis of granzyme B^+^ (I) and IFN-γ^+^ (J) T cells (*n *= 6 per group). (K) ­Schematic diagram illustrating how ICAM-1 enhances glycolysis through PI3K-AKT-mTOR signaling by interacting with LFA-1 on clustered T cells. All numerical data are presented as mean ± SD. *P* values were determined by an unpaired Student’s *t* test (B and H–J).

We have demonstrated that ICAM-1 upregulation following RT increases the quantity of tumor-infiltrating T cells, thereby contributing to elevated ^18^F-FDG uptake in tumors. Beyond this quantitative effect, we sought to determine whether ICAM-1 also regulates the metabolic activity of individual T cells to enhance their glycolytic capacity. To address this, T cells were isolated from the spleens of WT or *Icam1*-KO mice, and their *in vitro*  ^18^F-FDG uptake was measured (Fig. 6A). Compared to WT T cells, *Icam1*-KO T cells exhibited significantly reduced ^18^F-FDG uptake (Fig. 6B). Consistent with this, *Icam1*-KO T cells showed significantly reduced expression of the glucose transporters GLUT1 and GLUT3 (Fig. 6C), which mediate glucose uptake in activated T cells under transcriptional regulation by hypoxia-inducible factor-1α (Beckermann et al., 2020; Macintyre et al., 2014). In contrast, *Icam1*-OE T cells displayed markedly increased ^18^F-FDG uptake and GLUT1 and GLUT3 expression compared to WT controls (Fig. S20A and S20B), supporting a role for ICAM-1 in promoting T cell glucose metabolism. Inhibition of the ICAM-1–LFA-1 interaction using A-286982 also reduced ^18^F-FDG uptake and GLUT1 and GLUT3 expression in T cells (Fig. S20C and S20D), indicating that ICAM-1 promotes T cell glycolysis, at least in part, through interactions with LFA-1.

To further elucidate the metabolic consequences of ICAM-1 deficiency, we performed targeted metabolomic profiling of WT and *Icam1*-KO T cells. ICAM-1 deficiency led to a significant reduction in metabolites involved in glycolysis and the tricarboxylic acid (TCA) cycle (Fig. 6D), along with decreased abundance of multiple amino acids (Fig. S21), underscoring the broad impact of ICAM-1 on T cell metabolic activity. To explore the underlying mechanisms, RNA sequencing (RNA-seq) analysis was performed on WT and *Icam1*-KO T cells stimulated with anti-CD3/CD28. KEGG pathway analysis revealed dysregulation of key signaling pathways, including Th1/Th2/Th17 differentiation, FoxO, JAK-STAT, and mTOR signaling (Fig. S22). Since PI3K-AKT-mTOR signaling is essential for aerobic glycolysis and T cell growth upon TCR/CD28 co-stimulation (Buck et al., 2015), we hypothesized that ICAM-1 regulates glycolytic reprogramming via this pathway. Gene set enrichment analysis (GSEA) confirmed significant enrichment of PI3K-AKT and mTOR signaling in WT T cells (Fig. 6E and 6F). In agreement, western blot analysis showed that ICAM-1 deficiency or blockade of ICAM-1–LFA-1 interaction markedly reduced phosphorylation of PI3K, AKT, and mTOR, whereas ICAM-1 overexpression enhanced their phosphorylation (Figs. 6G, S23A and S23B).

Given the importance of interleukin-2 (IL-2) in T cell activation and survival (Cho et al., 2013; Dutta et al., 2017), GSEA also revealed enrichment of TCR and IL-2 signaling in WT T cells (Fig. S24A and S24B). Compared with WT cells, *Icam1*-KO T cells showed impaired proliferation (Fig. 6H) and reduced expression of effector molecules, including granzyme B and interferon-γ (IFN-γ), at both protein (Fig. 6I and 6J) and mRNA levels (Fig. S25A and S25B). Similarly, inhibition of ICAM-1–LFA-1 interaction significantly decreased granzyme B and IFN-γ expression (Fig. S26A and S26B), whereas ICAM-1 overexpression enhanced their production (Fig. S26C and S26D). Together, these findings demonstrate that ICAM-1 plays a critical role in T cell activation and metabolic reprogramming. Specifically, ICAM-1 promotes glycolysis and the TCA cycle in T cells via the PI3K-AKT-mTOR pathway, thereby enhancing glucose metabolism and ^18^F-FDG uptake (Fig. 6K).

## Discussion

Noninvasive evaluation of tumor responses to RT using ^18^F-FDG PET is essential for guiding personalized treatment strategies, including adjustments to radiation dose, fractionation, timing, and selection of optimal combination therapies (Bussink et al., 2011). However, RT-induced metabolic flares of ^18^F-FDG may complicate early response assessment and lead to potential misinterpretation of therapeutic efficacy (Ben-Haim and Ell, 2009; Weber, 2005). Understanding the mechanisms underlying these flares is critical for improving patient management. While prior studies have suggested that infiltration of inflammatory cells, such as neutrophils, macrophages, and lymphocytes, contributes to ^18^F-FDG flares after RT (Ben-Haim and Ell, 2009; Haberkorn et al., 1991), the specific mechanisms remain incompletely understood. Given that both inflammatory and residual tumor cells exhibit high ^18^F-FDG avidity, conventional PET imaging cannot distinguish between them. In this study, we provide evidence that ICAM-1^+^ T cells, rather than myeloid cells, are the primary mediators of tumor glucose metabolism following RT. Mechanistically, ICAM-1 enhances the effector function of T cells by activating the PI3K-AKT-mTOR signaling pathway and promoting the glycolytic activity of tumor-infiltrating T cells, thereby increasing their avidity for ^18^F-FDG. This T cell-intrinsic regulatory mechanism of ICAM-1 is a major contributor to the ^18^F-FDG PET flares observed after RT.

ICAM-1 is best recognized for mediating adhesion between antigen-presenting cells (such as DCs and macrophages) or endothelial cells and T cells via interaction with LFA-1 (Bui et al., 2020; Qian et al., 2024). This interaction promotes the formation of the immunological synapse between antigen-presenting cells and T cells, facilitates antigen presentation, and enables effective T cell activation and migration (Ramos et al., 2014; Zhang et al., 2022). In our study, the use of *Icam1*-KO mice revealed significantly reduced ^18^F-FDG uptake in tumors post-RT, highlighting the importance of host ICAM-1 in modulating tumor glucose metabolism. To specifically examine the role of T cell-intrinsic ICAM-1, we adoptively transferred *Icam1*-KO or *Icam1*-OE OT-I T cells. ICAM-1 expression on T cells was found to be both necessary and sufficient to promote T cell infiltration and increase ^18^F-FDG tumor uptake. Furthermore, pharmacologic inhibition of the ICAM-1–LFA-1 interaction impaired T cell infiltration, activation, and ^18^F-FDG uptake in tumors, suggesting that ICAM-1 facilitates T cell clustering through ICAM-1–LFA1 binding, promoting metabolic reprogramming in the TME.

The molecular mechanisms by which RT induces ICAM-1 upregulation on T cells remain incompletely understood. Previous studies have shown that ICAM-1 expression on endothelial cells is increased by tumor necrosis factor (TNF)-α through activation of the NF-κB signaling pathway (Qian et al., 2024). RT is known to stimulate the release of multiple cytokines within the TME, with their profiles shaped by radiation dose and fractionation. Moreover, accumulating evidence indicates that RT extensively remodels the immune landscape, enhancing the infiltration of various immune cell subsets and promoting cytokine secretion that drives metabolic rewiring in these cells (Galluzzi et al., 2023). Whether and how these RT-induced cytokine and metabolic cues converge to regulate ICAM-1 expression specifically on T cells remains an important question for future investigation.

Each immune cell subset within the TME exhibits distinct metabolic phenotypes. For example, M1 macrophages predominantly rely on glycolysis, whereas M2 macrophages preferentially utilize oxidative phosphorylation (OXPHOS) (Kolliniati et al., 2022; McLaughlin et al., 2020). T cell metabolism is similarly dynamic: upon activation through tumor-associate antigen recognition and co-stimulatory signals, T cells undergo metabolic reprogramming characterized by upregulated glycolysis, TCA cycle activity, and OXPHOS to support proliferation and effector function (Geltink et al., 2018; Leone and Powell, 2020). Notably, activated T cells often demand more glucose than tumor cells to sustain their function (Reinfeld et al., 2021). Our data identify ICAM-1 as a key regulator of this metabolic shift. Specifically, ICAM-1 deficiency impairs glycolysis and TCA cycle activity in T cells, in parallel with downregulation of the PI3K-AKT-mTOR pathway. These findings suggest that upregulating ICAM-1 expression may offer a strategy to metabolically reprogram T cells and enhance their antitumor activity. Indeed, we previously demonstrated that pharmacological upregulation of ICAM-1 augments systemic antitumor immunity when combined with RT (Zhao et al., 2021).

Given the central role of effector T cell infiltration in driving ^18^F-FDG PET flares, there is an urgent need for noninvasive imaging tools capable of distinguishing true tumor progression from immune-mediated inflammation. We previously demonstrated that PET imaging of granzyme B offers a noninvasive readout of T cell effector function in cancer patients (Shen et al., 2024; Zhou et al., 2022). However, since granzyme B is a secreted protein, longitudinal imaging is required to capture dynamic changes in its tumor expression during RT. In this study, we identified ICAM-1 as a cell-surface marker of metabolically active, tumor-infiltrating T cells. Thus, PET imaging of ICAM-1 expression may provide a means to assess both T cell functionality and metabolic activity within the TME following RT. When used in conjunction with conventional ^18^F-FDG PET, ICAM-1-targeted PET imaging could improve response classification by differentiating true progression from pseudoprogression or clinical response (Fig. S27), thereby addressing the diagnostic limitations of ^18^F-FDG PET alone.

In summary, our findings reveal ICAM-1 as a key regulator of T cell glucose metabolism and intratumoral infiltration in response to RT. By promoting LFA-1-mediated interactions, ICAM-1 enhances T cell effector function and ^18^F-FDG uptake, contributing to RT-induced metabolic flares. ICAM-1-targeted PET imaging may therefore serve as a valuable tool to monitor immune dynamics in the TME, offering a more accurate evaluation of RT efficacy and supporting the rational design of combinational therapy strategies.

## Supplementary Material

pwaf111_Supplementary_Data

## Data Availability

All data generated and supporting the findings of this study are available within the main text and/or supplementary information. Further information and materials will be made available upon reasonable request from the corresponding author (Z.L.).

## References

[pwaf111-B1] Abdel-Wahab M , Giammarile F, Carrara M et al. Radiotherapy and theranostics: a lancet oncology commission. Lancet Oncol 2024;25:e545–e580. 39362232 10.1016/S1470-2045(24)00407-8

[pwaf111-B2] Bai JW , Qiu SQ, Zhang GJ. Molecular and functional imaging in cancer-targeted therapy: current applications and future directions. Signal Transduct Target Ther 2023;8:89. 36849435 10.1038/s41392-023-01366-y PMC9971190

[pwaf111-B3] Basu S , Alavi A. Defining co-related parameters between ‘metabolic’ flare and ‘clinical’, ‘biochemical’, and ‘osteoblastic’ flare and establishing guidelines for assessing response to treatment in cancer. Eur J Nucl Med Mol Imaging 2007;34:441–443. 17072613 10.1007/s00259-006-0264-6

[pwaf111-B4] Beckermann KE , Hongo R, Ye X et al. CD28 costimulation drives tumor-infiltrating T cell glycolysis to promote inflammation. JCI Insight 2020;5:e138729. 32814710 10.1172/jci.insight.138729 PMC7455120

[pwaf111-B5] Ben-Haim S , Ell P. ^18^F-FDG PET and PET/CT in the evaluation of cancer treatment response. J Nucl Med 2009;50:88–99. 19139187 10.2967/jnumed.108.054205

[pwaf111-B6] Buck MD , O’sullivan D, Pearce EL. T cell metabolism drives immunity. J Exp Med 2015;212:1345–1360. 26261266 10.1084/jem.20151159 PMC4548052

[pwaf111-B7] Bui TM , Wiesolek HL, Sumagin R. ICAM-1: a master regulator of cellular responses in inflammation, injury resolution, and tumorigenesis. J Leukoc Biol 2020;108:787–799. 32182390 10.1002/JLB.2MR0220-549R PMC7977775

[pwaf111-B8] Bussink J , Kaanders JH, van der Graaf WT et al. PET-CT for radiotherapy treatment planning and response monitoring in solid tumors. Nat Rev Clin Oncol 2011;8:233–242. 21263464 10.1038/nrclinonc.2010.218

[pwaf111-B9] Chandra RA , Keane FK, Voncken FEM et al. Contemporary radiotherapy: present and future. Lancet 2021;398:171–184. 34166607 10.1016/S0140-6736(21)00233-6

[pwaf111-B10] Cho JH , Kim HO, Kim KS et al. Unique features of naive CD8^+^ T cell activation by IL-2. J Immunol 2013;191:5559–5573. 24166977 10.4049/jimmunol.1302293

[pwaf111-B11] Dustin ML. Integrins and their role in immune cell adhesion. Cell 2019;177:499–501. 30952447 10.1016/j.cell.2019.03.038

[pwaf111-B12] Dutta D , Barr VA, Akpan I et al. Recruitment of calcineurin to the TCR positively regulates T cell activation. Nat Immunol 2017;18:196–204. 27941787 10.1038/ni.3640 PMC6352896

[pwaf111-B13] Galluzzi L , Aryankalayil MJ, Coleman CN et al. Emerging evidence for adapting radiotherapy to immunotherapy. Nat Rev Clin Oncol 2023;20:543–557. 37280366 10.1038/s41571-023-00782-x

[pwaf111-B14] Geltink RIK , Kyle RL, Pearce EL. Unraveling the complex interplay between T cell metabolism and function. Annu Rev Immunol 2018;36:461–488. 29677474 10.1146/annurev-immunol-042617-053019 PMC6323527

[pwaf111-B15] Haberkorn U , Strauss LG, Dimitrakopoulou A et al. PET studies of fluorodeoxyglucose metabolism in patients with recurrent colorectal tumors receiving radiotherapy. J Nucl Med 1991;32:1485–1490. 1714497

[pwaf111-B16] Herrera FG , Bourhis J, Coukos G. Radiotherapy combination opportunities leveraging immunity for the next oncology practice. CA Cancer J Clin 2017;67:65–85. 27570942 10.3322/caac.21358

[pwaf111-B17] Ito M , Komai K, Mise-Omata S et al. Brain regulatory T cells suppress astrogliosis and potentiate neurological recovery. Nature 2019;565:246–250. 30602786 10.1038/s41586-018-0824-5

[pwaf111-B18] Kolliniati O , Ieronymaki E, Vergadi E et al. Metabolic regulation of macrophage activation. J Innate Immun 2022;14:51–68. 34247159 10.1159/000516780 PMC8787535

[pwaf111-B19] Leone RD , Powell JD. Metabolism of immune cells in cancer. Nat Rev Cancer 2020;20:516–531. 32632251 10.1038/s41568-020-0273-y PMC8041116

[pwaf111-B20] Macintyre AN , Gerriets VA, Nichols AG et al. The glucose transporter Glut1 is selectively essential for CD4 T cell activation and effector function. Cell Metab 2014;20:61–72. 24930970 10.1016/j.cmet.2014.05.004 PMC4079750

[pwaf111-B21] Mclaughlin M , Patin EC, Pedersen M et al. Inflammatory microenvironment remodelling by tumour cells after radiotherapy. Nat Rev Cancer 2020;20:203–217. 32161398 10.1038/s41568-020-0246-1

[pwaf111-B22] Pijl JP , Nienhuis PH, Kwee TC et al. Limitations and pitfalls of FDG-PET/CT in infection and inflammation. Semin Nucl Med 2021;51:633–645. 34246448 10.1053/j.semnuclmed.2021.06.008

[pwaf111-B23] Qian WJ , Yan JS, Gang XY et al. Intercellular adhesion molecule-1 (ICAM-1): from molecular functions to clinical applications in cancer investigation. Biochim Biophys Acta Rev Cancer 2024;1879:189187. 39317271 10.1016/j.bbcan.2024.189187

[pwaf111-B24] Rahman WT , Wale DJ, Viglianti BL et al. The impact of infection and inflammation in oncologic ^18^F-FDG PET/CT imaging. Biomed Pharmacother 2019;117:109168. 31334700 10.1016/j.biopha.2019.109168 PMC7104808

[pwaf111-B25] Ramos TN , Bullard DC, Barnum SR. ICAM-1: isoforms and phenotypes. J Immunol 2014;192:4469–4474. 24795464 10.4049/jimmunol.1400135 PMC4015451

[pwaf111-B26] Reinfeld BI , Madden MZ, Wolf MM et al. Cell-programmed nutrient partitioning in the tumour microenvironment. Nature 2021;593:282–288. 33828302 10.1038/s41586-021-03442-1 PMC8122068

[pwaf111-B27] Rodríguez-Ruiz ME , Vanpouille-Box C, Melero I et al. Immunological mechanisms responsible for radiation-induced abscopal effect. Trends Immunol 2018;39:644–655. 30001871 10.1016/j.it.2018.06.001 PMC6326574

[pwaf111-B28] Rodriguez-Ruiz ME , Vitale I, Harrington KJ et al. Immunological impact of cell death signaling driven by radiation on the tumor microenvironment. Nat Immunol 2020;21:120–134. 31873291 10.1038/s41590-019-0561-4

[pwaf111-B29] Sabatos CA , Doh J, Chakravarti S et al. A synaptic basis for paracrine interleukin-2 signaling during homotypic T cell interaction. Immunity 2008;29:238–248. 18674934 10.1016/j.immuni.2008.05.017 PMC4466225

[pwaf111-B30] Shen X , Zhou H, Zhou X et al. ^68^Ga-grazytracer PET for noninvasive assessment of response to immunotherapy in solid tumors and lymphomas: a phase 1/2 clinical trial. Nat Commun 2024;15:8791. 39389969 10.1038/s41467-024-53197-2 PMC11467221

[pwaf111-B31] van Baardwijk A , Bosmans G, Dekker A et al. Time trends in the maximal uptake of FDG on PET scan during thoracic radiotherapy. A prospective study in locally advanced non-small cell lung cancer (NSCLC) patients. Radiother Oncol 2007;82:145–152. 17258339 10.1016/j.radonc.2007.01.007

[pwaf111-B32] van Heertum RL , Scarimbolo R, Wolodzko JG et al. Lugano 2014 criteria for assessing FDG-PET/CT in lymphoma: an operational approach for clinical trials. Drug Des Devel Ther 2017;11:1719–1728. 10.2147/DDDT.S136988 PMC5479259 28670108

[pwaf111-B33] Vander Heiden MG , Deberardinis RJ. Understanding the intersections between metabolism and cancer biology. Cell 2017;168:657–669. 28187287 10.1016/j.cell.2016.12.039 PMC5329766

[pwaf111-B34] Wahl RL , Jacene H, Kasamon Y et al. From RECIST to PERCIST: evolving considerations for PET response criteria in solid tumors. J Nucl Med, 2009;50 Suppl 1:122s–150s. 19403881 10.2967/jnumed.108.057307 PMC2755245

[pwaf111-B35] Weber WA. Use of PET for monitoring cancer therapy and for predicting outcome. J Nucl Med 2005;46:983–995. 15937310

[pwaf111-B36] Weber WA. Assessing tumor response to therapy. J Nucl Med 2009;50 Suppl 1:1s–10s. 19380403 10.2967/jnumed.108.057174

[pwaf111-B37] Wei Y , Guo J, Lu N et al. Magnesium enhances the graft-versus-tumor effect of donor lymphocytic infusion on hematologic malignancies. Hematol Oncol 2023;41:922–932. 37496287 10.1002/hon.3207

[pwaf111-B38] Zhang T , Zhang Y, Zhao Y et al. Annotation of CD8^+^ T-cell function via ICAM-1 imaging identifies FAK inhibition as an adjuvant to augment the antitumor immunity of radiotherapy. *Theranostics* 2024;14:699–713. 38169608 10.7150/thno.90709 PMC10758046

[pwaf111-B39] Zhang W , Zhong W, Wang B et al. ICAM-1-mediated adhesion is a prerequisite for exosome-induced T cell suppression. Dev Cell 2022;57:329–343.e7. 35085484 10.1016/j.devcel.2022.01.002 PMC8881799

[pwaf111-B40] Zhao Y , Zhang T, Wang Y et al. ICAM-1 orchestrates the abscopal effect of tumor radiotherapy. Proc Natl Acad Sci U S A 2021;118:e2010333118. 33785590 10.1073/pnas.2010333118 PMC8040592

[pwaf111-B41] Zhou H , Wang Y, Xu H, et al. Noninvasive interrogation of CD8^+^ T cell effector function for monitoring early tumor responses to immunotherapy. *J Clin Invest* 2022;132:e161065. 35788116 10.1172/JCI161065 PMC9374377

[pwaf111-B42] Zhou T , Damsky W, Weizman OE et al. IL-18BP is a secreted immune checkpoint and barrier to IL-18 immunotherapy. Nature 2020;583:609–614. 32581358 10.1038/s41586-020-2422-6 PMC7381364

