# Supplementary material for: ICAM-1 promotes T cell glycolytic reprogramming and tumor infiltration to drive 18F-FDG PET flares following radiotherapy
Source: Protein Cell. 2025 Dec 19;17(7):631–43. doi: 10.1093/procel/pwaf111 (PMC13340927; doi:10.1093/procel/pwaf111)
Supplement: pwaf111_Supplementary_Data [file pwaf111_supplementary_data.pdf]

Supplementary Information for

ICAM-1 promotes T cell glycolytic reprogramming and tumor infiltration to drive ^{18}F-FDG PET flares following radiotherapy

Rui Song[1†], Meixin Zhao[2†*], Ting Zhang[1†], Yining Zhang[1], Fuxin Guo[3], Huiying He[4], Haoyi Zhou[1], Kui Li[1], Jianze Wang[1], Jinhong Du[1], Feng Wang[1,5], Shixin Zhou[6], Hua Zhu[1,5], Jiadong Wang[1], Weifang Zhang[2*], Zhi Yang[1,5*], Zhaofei Liu[1,2,5*]

[1]Department of Nuclear Medicine, Peking University Cancer Hospital and Department of Radiation Medicine, School of Basic Medical Sciences, Peking University, Beijing, China

[2]State Key Laboratory of Vascular Homeostasis and Remodeling, Department of Nuclear Medicine, Peking University Third Hospital, Beijing, China

[3]Department of Radiation Oncology, Peking University Third Hospital, Beijing, China

[4]Department of Pathology, Peking University Third Hospital, Beijing, China

[5]Key Laboratory of Carcinogenesis and Translational Research (Ministry of Education/Beijing), NMPA Key Laboratory for Research and Evaluation of Radiopharmaceuticals (National Medical Products Administration), Peking University Cancer Hospital, Beijing, China

[6]Department of Cell Biology, School of Basic Medical Sciences, Peking University, Beijing, China

[†]These authors contributed equally to this work.

*Corresponding authors: Zhaofei Liu, Email: liuzf@bjmu.edu.cn; Zhi Yang, Email: pekyz@163.com; Weifang Zhang, Email: tsy1997@126.com; Meixin Zhao, Email: zhaomeixin.student@sina.com

This file includes:

Materials and Methods

Figures S1 to S27

Tables S1 to S3

Materials and Methods

Study approval

All animal experiments were conducted in accordance with the protocols approved by the Institutional Animal Care and Use Committee at Peking University. Clinical PET/CT scanning and studies involving human tumor samples were approved by the Institutional Review Board of Peking University Third Hospital. All patient samples were de-identified prior to use in this study.

Clinical ^{18}F-FDG PET/CT imaging and immunohistochemistry

A retrospective analysis was conducted using routine clinical ^{18}F-FDG PET/CT scans of patients before and after RT at Peking University Third Hospital. Two representative cases were selected for inclusion in this study. Detailed patient demographics are provided in Table S1.

Tumor specimens obtained before and after RT were collected from Peking University Third Hospital. Information regarding RT dosage, treatment timelines, and sample collection time points is summarized in Table S2. For immunohistochemistry, formalin-fixed, paraffin-embedded tumor sections underwent antigen retrieval and were incubated overnight at 4°C with primary antibodies, followed by incubation with horseradish peroxidase (HRP)-conjugated secondary antibodies for 2 h at room temperature. Staining was visualized using diaminobenzidine (DAB) and examined under an Eclipse Ci-E microscope (Nikon, Tokyo, Japan). The following primary antibodies were used: anti-human ICAM-1 (1:200; Abcam, Cambridge, MA), anti-human CD3 (1:200; Abcam), anti-human CD11b (1:2000; Abcam), and anti-human CD31 (1:50; Abcam).

Cell lines and animal models

The MC38 and MC38-OVA murine colon carcinoma, Lewis lung carcinoma, and 4T1 breast cancer cell lines were obtained from the American Type Culture Collection (ATCC). MC38, MC38-OVA, and Lewis lung carcinoma cells were cultured in Dulbecco’s modified Eagle medium (DMEM; Invitrogen, Carlsbad, CA), while 4T1 cells were cultured in RPMI 1640 medium (Invitrogen). All cells were grown in a medium supplemented with 10% fetal bovine serum and maintained at 37°C in a humidified atmosphere containing 5% CO_2.

Female WT (CD45.2$^+$) C57BL/6 and CD45.1$^+$ C57BL/6 (B6.SJL-*Ptprc*^{a}

Pepc^b/BoyCrl) mice were obtained from the Department of Laboratory Animal Science at Peking University. OT-I transgenic (C57BL/6-Tg (TcraTcrb)1100Mjb/J) and *Icam1*-KO (C57BL/6-*Icam1^em1Smoc^*) C57BL/6 mice were purchased from Shanghai Model Organisms Center (Shanghai, China). *Icam1*-KO OT-I mice were generated by crossing OT-I mice with *Icam1*-KO mice. To establish tumor-bearing mouse models, 1×10^6 tumor cells were subcutaneously injected into the right flank of mice. Tumor size was measured every other day with calipers and calculated using the formula: volume = length × width^2/2.

Radiotherapy

MC38 tumor- or Lewis lung carcinoma-bearing WT or *Icam1*-KO C57BL/6 mice were randomly divided into control and RT groups. Tumors in the RT group were irradiated using an X-ray irradiator (RS2000 PRO, 160 kV, 25 mA; Rad Source Technologies, Suwanee, GA). Irradiation was targeted to the tumor region with a 5-mm margin and delivered as a total of 20 Gy in two fractions of 10 Gy, administered every other day.

Preparation of ^{89}Zr-DFO-αICAM-1/Fab

The ICAM-1-specific PET radiotracer ^{89}Zr-DFO-αICAM-1/Fab was synthesized as previously described (Zhao et al., 2017, Lai et al., 2018). Briefly, the anti-ICAM-1 antibody (αICAM-1; clone YN1/1.7.4; BioXcell, West Lebanon, NH) was digested into Fab fragments using a Fab preparation kit (Thermo Fisher Scientific, Waltham, MA). The resulting Fab (αICAM-1/Fab) was conjugated with the bifunctional chelator *p*-SCN-Bn-deferoxamine (DFO; Macrocyclics, Dallas, TX) at a molar ratio of 1:10 in $NaHCO_3$ buffer (pH 8.5–9.0).

For radiolabeling, ^{89}Zr-oxalic acid was neutralized to pH 7.0 using 0.5 M HEPES and 1 M Na_2CO_3 buffer and then mixed with 50 µg of DFO-αICAM-1/Fab at 37 °C for 1 h with shaking. The resulting ^{89}Zr-DFO-αICAM-1/Fab was purified using a PD-10 desalting column (GE Healthcare, Piscataway, NJ), and the radiochemical purity was measured by instant thin-layer chromatography. A radiolabeled isotype control Fab (^{89}Zr-DFO-IgG/Fab) was prepared using the same procedure.

In vitro cell binding assay

The binding specificity of ^{89}Zr-DFO-αICAM-1/Fab was assessed in vitro using

ICAM-1-positive 4T1 cells. Cells cultured in 24-well plates were incubated with 18.5 kBq of ^{89}Zr-DFO-IgG/Fab or ^{89}Zr-DFO-αICAM-1/Fab with or without a 500-fold molar excess of unlabeled DFO-αICAM-1/Fab for blocking. After incubation at 4°C for 2 h, cells were washed with phosphate-buffered saline (PBS), collected, and measured for associated radioactivity using a γ-counter (Packard, Waltham, MA). Results are presented as the percentage of added dose per million cells (%AD/10^6 cells).

Small-animal PET imaging

Small-animal PET imaging and data analysis was performed using a Super Nova PET/CT scanner (Pingseng Healthcare, Shanghai, China). For ^{89}Zr-DFO-αICAM-1/Fab PET imaging, MC38 tumor-bearing mice received intravenous injection of 3.7 MBq of the radiotracer. 10-min static PET scans were acquired at 2, 6, 12, 24, and 48 h post-injection. ^{89}Zr-DFO-IgG/Fab PET imaging followed the same protocol.

For ^{18}F-FDG PET imaging, MC38, Lewis lung carcinoma, or MC38-OVA tumor-bearing mice (WT or *Icam1*-KO) were fasted overnight and anesthetized with isoflurane before receiving 5.55 MBq of ^{18}F-FDG via intravenous injection. A 10-min static PET scan was performed 45 min post-injection. Quantification was conducted by calculating region-of-interest (ROI)-derived %ID/g values as described previously (Feng et al., 2020).

Ex vivo ^{18}F-FDG uptake assay

MC38 tumor-bearing WT or *Icam1*-KO C57BL/6 mice were fasted overnight and intravenously injected with 37 MBq of ^{18}F-FDG. At 45 min post-injection, the mice were euthanized and tumors were harvested. Tumors were enzymatically dissociated into single-cell suspensions using 10 U/mL collagenase I, 400 U/mL collagenase IV, and 30 U/mL DNase (Yuanye Bio-Technology, Shanghai, China). Immune cell populations were isolated using antibody-conjugated magnetic microbeads targeting CD45, CD4/CD8, CD11b, Ly6G, CD11c, or F4/80 (Miltenyi Biotec, Teterow, Germany). Cells were incubated in 90 μL magnetic-activated cell sorting (MACS) buffer with 10 μL microbeads for 15 min and passed through LS columns (Miltenyi Biotec) using QuadroMACS Separators according to the manufacturer's protocol. The radioactivity associated with sorted cells was measured using a γ-counter.

In vivo ICAM-1 blockade and FTY720 treatment

To assess the role of ICAM-1 in ^{18}F-FDG tumor uptake, MC38 tumor-bearing mice received intraperitoneal injections of either PBS or 200 μg anti-ICAM-1 antibody (clone YN1/1.7.4; BioXcell) on days -2, 0, 2, and 4. To assess the impact of tumor-infiltrating T cells on ^{18}F-FDG uptake in tumors, MC38 tumor-bearing mice were administered 40 μg FTY720 (Cayman Chemical, Ann Arbor, MI) intraperitoneally on day -1, followed by daily injections of 20 μg from day 0 to day 8.

Isolation, culture, and proliferation assay of WT or *Icam1*-KO T cells

T cells were isolated from lymph nodes and spleens of WT or *Icam1*-KO mice using CD4/CD8 microbeads and LS columns (Miltenyi Biotec) according to the manufacturer's instructions. Cells were cultured in complete RPMI 1640 medium supplemented with IL-2 (10 ng/mL; Peprotech, Cranbury, NJ) and stimulated with plate-bound anti-CD3 (1 μg/mL) and anti-CD28 (2 μg/mL) antibodies (BioLegend, San Diego, CA). ICAM-1 deficiency in T cells from *Icam1*-KO mice was validated by flow cytometry.

For proliferation assays, T cells were labeled with 2.5 μM CFSE (Thermo Fisher Scientific) and activated in 24-well plates with anti-CD3 (1 μg/mL) and anti-CD28 (2 μg/mL) antibodies for 2 days. Afterwards, T cell proliferation was determined by flow cytometric analysis of CFSE.

Co-culture of BMDCs and T cells

The isolation, culture, and stimulation of BMDCs were performed as previously described (Inaba et al., 1992). Tumor tissues from MC38 tumor-bearing mice (control or RT group) were lysed to extract proteins, and protein concentrations were quantified using a bicinchoninic acid (BCA) protein assay kit (Thermo Fisher Scientific). Mature BMDCs were harvested after stimulation and co-cultured with T cells (1×10^5 BMDCs with 1×10^6 T cells) in the presence of tumor lysate (1 mg/mL). After 24 h, cells were collected for flow cytometric analysis of ICAM-1 expression.

Transwell migration assay

WT or *Icam1*-KO T cells (1×10^6 cells) were seeded in 5 μm-pore transwell inserts (Corning, Corning, NY) placed in 12-well plates containing RPMI 1640 medium. Tumor lysates (1 mg/mL), prepared from MC38 tumors collected from mice post-RT, were added to the lower chambers as chemoattractants. After 24 h of incubation, T cells

that migrated to the lower chambers were collected and counted.

T cell tracking assay

WT T cells isolated from mouse spleens were labeled with CFSE (Thermo Fisher Scientific) or DiR (AAT Bioquest, Sunnyvale, CA), and stimulated with anti-CD3 (1 μg/mL) and anti-CD28 (2 μg/mL) antibodies. For blocking studies, ICAM-1 was blocked with anti-ICAM-1 antibody (20 μg/mL) for 30 min at 4°C. MC38 tumor-bearing mice received 5×10^6 DiR- or CFSE-labeled T cells via tail vein injection. At 24 h post-injection, mice receiving DiR-labeled T cells underwent near-infrared fluorescence imaging (excitation wavelength, 745 nm; emission wavelength, 800 nm) using an IVIS Spectrum In Vivo Imaging System (Xenogen, Alameda, CA). For CFSE-labeled T cells, tumors were harvested 24 h post-injection for immunofluorescence staining.

Retroviral transduction

MACS-purified T cells from WT C57BL/6 or OT-I transgenic mice were cultured for 36 h and then transduced with either control or *Icam1*-overexpressing (*Icam1*-OE) retrovirus by spin infection at 1700 rpm for 2 h in the presence of polybrene (8 μg/mL; Yeasen Biotechnology, Shanghai, China). Following transduction, ICAM-1 expression was determined by flow cytometry and immunofluorescence staining.

In vitro T cell uptake of ^{18}F-FDG

WT or *Icam1*-KO T cells were cultured in complete RPMI 1640 medium for 48 h. For blocking studies, the ICAM-1–LFA-1 interaction inhibitor A-286982 (500 nM; MedChemExpress, Monmouth Junction, NJ) was added during culture. Cells were then seeded in 48-well plates at a density of 4×10^5 cells per well and incubated in glucose-free RPMI 1640 medium (Invitrogen) for 12 h to induce metabolic starvation. Subsequently, cells were incubated with ^{18}F-FDG (111 kBq/well) at 37°C for 2 h, washed with cold PBS, and harvested. Cell-associated radioactivity was measured using a γ-counter.

Adoptive transfer of OT-I T cells

Single-cell suspensions isolated from the lymph nodes and spleens of CD45.2$^+$ WT or *Icam1*-KO OT-I transgenic mice were subjected to MACS using the mouse CD8$^+$ T

Cell Isolation Kit (Miltenyi Biotec), following the manufacturer's instructions. Purified WT or *Icam1*-KO OT-I T cells were cultured in complete RPMI 1640 medium supplemented with IL-2 (10 ng/mL) and stimulated with $OVA_{257–264}$ peptide (1 μM; Yuanye Bio-Technology) for 2 days prior to adoptive transfer.

To investigate the role of ICAM-1–LFA-1 interactions, OT-I T cells were pre-treated with A-286982 (500 nM). To further assess the contribution of ICAM-1 on T cells, OT-I cells were transduced with either control or *Icam1*-OE retrovirus. MC38-OVA tumor-bearing $CD45.1^+$ C57BL/6 mice were intravenously injected with 2×10^6 OT-I T cells when tumor volumes reached approximately 200 mm^3. Tumor growth was monitored every other day, and ^{18}F-FDG PET imaging was performed on days 0 and 4. On day 4, tumors were harvested for flow cytometric analysis of $CD45.2^+$ T cell infiltration.

In a separate experiment, $CD45.2^+$ WT and *Icam1*-KO OT-I T cells were mixed at a 1:1 ratio and adoptively transferred into MC38-OVA tumor-bearing $CD45.1^+$ C57BL/6 mice via tail vein. Tumors were collected 4 days post-transfer for flow cytometric analysis of $CD45.2^+$ cell populations.

Metabolomic analysis

Metabolomic analysis was performed as previously described (Zhou et al., 2019). Briefly, WT or *Icam1*-KO T cells were activated in 24-well plates using mouse anti-CD3 and anti-CD28 antibodies for 2 days. Metabolites were extracted using a chloroform:methanol solution (2:1, v/v) and analyzed with an Ultimate 3000 UHPLC system coupled to a Q-Exactive HF mass spectrometer (Thermo Fisher Scientific). Targeted metabolite quantification was conducted using data-dependent acquisition (DDA) and parallel reaction monitoring (PRM) modes. Raw data from DDA and PRM analyses were processed with MS-DIAL and Skyline software, and further filtered and analyzed using MetaboAnalyst 5.0.

RNA sequencing analysis

Total RNA was extracted with TRIzol reagent (Invitrogen) from WT or *Icam1*-KO T cells, and RNA libraries were prepared and sequenced on the BGISEQ-500 platform at the Beijing Genomics Institute. Raw sequencing data were processed through a standard RNA-seq analysis pipeline. Clean reads were aligned to the reference genome and gene set using HISAT2 and Bowtie2, and transcript abundance was quantified and

normalized with RSEM. Differential expression analysis was performed with the DESeq2 package in R, applying a significance cutoff of adjusted $P < 0.05$ and an absolute fold change > 2.

Real-time polymerase chain reaction (PCR)

Total RNA was extracted from WT or *Icam1*-KO T cells using the RNA-Quick Purification Kit (Beyotime Biotechnology, Shanghai, China), and reverse-transcribed into cDNA using a cDNA synthesis kit (Applied Biological Materials, Richmond, Canada). Quantitative real-time PCR was performed using Hieff® qPCR SYBR Green Master Mix (Yeasen Biotechnology) on a QuantStudio™ 6 Flex Real-Time PCR System (Applied Biosystems, Wilmington, DE). Relative mRNA expression levels were calculated using the $2^{-\Delta\Delta CT}$ method. The primer sequences used were as follows: *Gapdh* forward: TTGATGGCAACAATCTCCAC; *Gapdh* reverse: CGTCCCGTAGACAAAATGGT; *Ifng* forward: ACAGCAAGGCGAAAAAGGATG; *Ifng* reverse: TGGTGGACCACTCGGATGA; and *Gzmb* forward: GCTGCTAAAGCTGAAGAGT; *Gzmb* reverse: ACATAGCACACATCTCCTG.

Western blotting

Cells were lysed on ice in RIPA buffer (Thermo Fisher Scientific) supplemented with a protease inhibitor cocktail (1:100; Yamei, Shanghai, China) and a phosphatase inhibitor cocktail (1:100; Yamei). Protein concentrations were determined using a BCA Protein Assay Kit (Thermo Fisher Scientific). Equal amounts of protein were separated by SDS-PAGE and transferred onto polyvinylidene difluoride (PVDF) membranes. After blocking with 5% bovine serum albumin (BSA), membranes were incubated with primary antibodies overnight at 4°C, followed by incubation with HRP-conjugated species-specific secondary antibodies. The primary and secondary antibodies used were as follows: anti-mouse GLUT1 (1:1000; Immunoway, Plano, TX), anti-mouse GLUT3 (1:1000; Immunoway), anti-mouse PI3K p85 alpha (1:1,000; Abcam), anti-mouse phosphorylated PI3K p85 alpha (1:1,000, Abcam), anti-mouse AKT (1:10,000; Abcam), anti-mouse phosphorylated AKT (1:1,000; Abcam), anti-mouse mTOR (1:10,000, Abcam), anti-mouse phosphorylated mTOR (1:1000; Immunoway), mouse anti-β-actin (1:10,000; Beyotime Biotechnology), HRP-conjugated goat anti-mouse IgG (1:10,000; Beyotime Biotechnology), and HRP-conjugated goat anti-rabbit IgG (1:10,000; Beyotime Biotechnology).

Flow cytometric analysis

Tumor tissues harvested from tumor-bearing mice were enzymatically digested into single-cell suspensions and subjected to flow cytometric analysis using a Cytek Aurora flow cytometer (Cytek Biosciences, Fremont, CA). For intracellular staining of granzyme B and IFN-γ, cells were first stimulated with a cell activation cocktail (BioLegend), followed by fixation and permeabilization using the Permeabilization Wash Buffer (BioLegend). Cells were then stained with fluorescently labeled antibodies. The complete list of antibodies used is provided in Table S3.

Immunofluorescence staining

For immunofluorescence staining of ICAM-1 and LFA-1 on T cells, cells cultured on 20 mm glass-bottom dishes were fixed with 4% paraformaldehyde, blocked with 5% BSA, and incubated overnight at 4°C with anti-mouse ICAM-1 (1:100; Santa Cruz; Dallas, TX) and anti-mouse LFA-1 (1:100; Abclonal, Wuhan, China). After washing with PBS, the cells were incubated with FITC- or DyLight549-conjugated secondary antibodies (1:200; EarthOx, Millipore, CA) for 1 h, followed by nuclear counterstaining with DAPI (Solarbio, Beijing, China) for 15 min. Images were acquired using a confocal microscope (Leica, Wetzlar, Germany).

To visualize T cell cluster formation, WT T cells with or without pre-treatment with A-286982 (500 nM), or *Icam1*-KO T cells, were seeded on 20 mm glass-bottom dishes and labeled with CFSE. T cell-T cell interactions and cluster formation were examined using confocal microscopy.

For immunofluorescence staining of tumor tissues, frozen MC38 tumor sections were fixed with ice-cold acetone, blocked with 5% BSA, and incubated overnight at 4°C with anti-mouse ICAM-1 (1:100; Santa Cruz) or anti-mouse CD3 (1:100; Abcam). Sections were subsequently incubated with DyLight549-conjugated secondary antibodies (1:200; EarthOx) and counterstained with DAPI for 1 h before imaging with a confocal microscope.

Statistical analyses

Statistical analyses were performed using GraphPad Prism version 9.0 (GraphPad Software, San Diego, CA). Quantitative data are presented as mean ± standard deviation (SD). Comparisons between two independent groups were conducted using a two-tailed

unpaired Student's *t* test. For comparisons of ICAM-1, CD3, CD11b, and CD31 expression before and after RT within the same individual, a paired Student's *t* test was used. Differences in cell binding assay and ^{18}F-FDG tumor uptake among multiple groups were analyzed using one-way analysis of variance (ANOVA) followed by Tukey's post hoc test. Two-way ANOVA was employed to assess tumor growth over time. Pearson's correlation analysis was performed to assess the relationship between two variables. A P value < 0.05 was considered statistically significant.

References

FENG, X., WANG, Y., LU, D., XU, X., ZHOU, X., ZHANG, H., ZHANG, T., ZHU, H., YANG, Z., WANG, F., LI, N. & LIU, Z. (2020) Clinical translation of a 68Ga-labeled integrin αvβ6-targeting cyclic radiotracer for PET imaging of pancreatic cancer. *J Nucl Med,* 61**,** 1461-1467.

INABA, K., INABA, M., ROMANI, N., AYA, H., DEGUCHI, M., IKEHARA, S., MURAMATSU, S. & STEINMAN, R. M. (1992) Generation of large numbers of dendritic cells from mouse bone marrow cultures supplemented with granulocyte/macrophage colony-stimulating factor. *J Exp Med,* 176**,** 1693-702.

LAI, J., LU, D., ZHANG, C., ZHU, H., GAO, L., WANG, Y., BAO, R., ZHAO, Y., JIA, B., WANG, F., YANG, Z. & LIU, Z. (2018) Noninvasive small-animal imaging of galectin-1 upregulation for predicting tumor resistance to radiotherapy. *Biomaterials,* 158**,** 1-9.

ZHAO, Y., ZHANG, C., GAO, L., YU, X., LAI, J., LU, D., BAO, R., WANG, Y., JIA, B., WANG, F. & LIU, Z. (2017) Chemotherapy-induced macrophage infiltration into tumors enhances nanographene-based photodynamic therapy. *Cancer Res,* 77**,** 6021-6032.

ZHOU, J., SUN, L., CHEN, L., LIU, S., ZHONG, L. & CUI, M. (2019) Comprehensive metabolomic and proteomic analyses reveal candidate biomarkers and related metabolic networks in atrial fibrillation. *Metabolomics,* 15**,** 96.

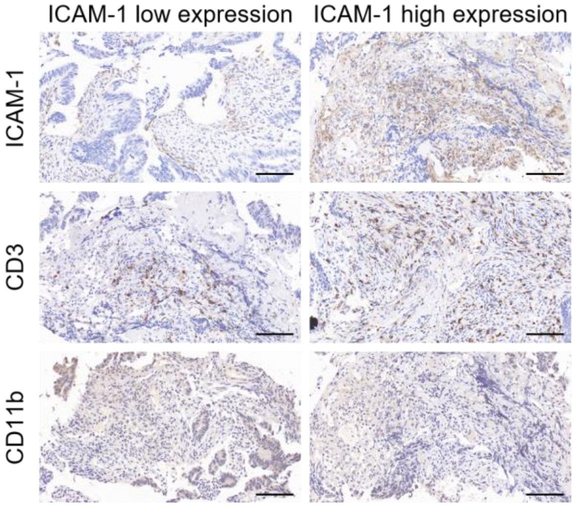

Figure S1. Immunohistochemical staining of tumor tissues. Immunohistochemical staining of ICAM-1, CD3, and CD11b in ICAM-1 low- and high-expressing regions of the post-RT tumor from patient #2 (Table S1). Scale, 100 μm.

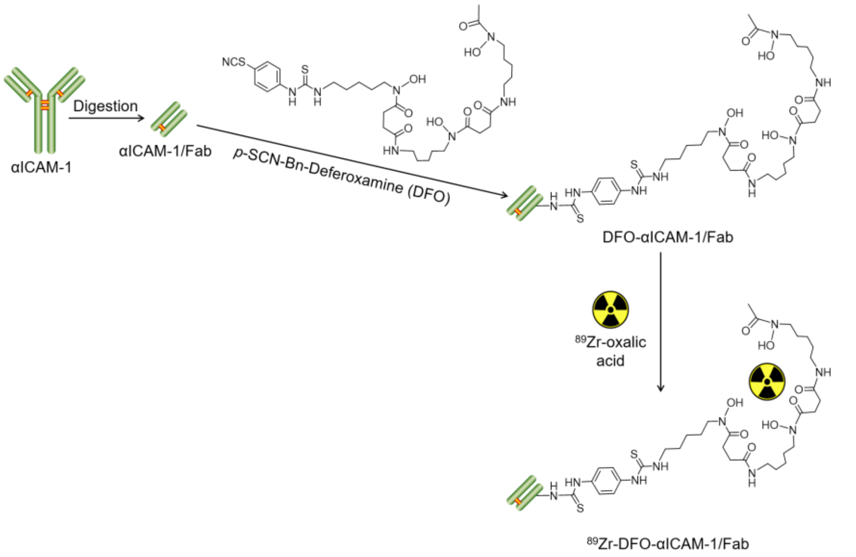

Figure S2. Synthetic scheme of ^{89}Zr-DFO-αICAM-1/Fab. The Fab fragment of αICAM-1 (αICAM-1/Fab) was conjugated with deferoxamine (DFO), followed by radiolabeling with ^{89}Zr-oxalic acid to obtain ^{89}Zr-DFO-αICAM-1/Fab.

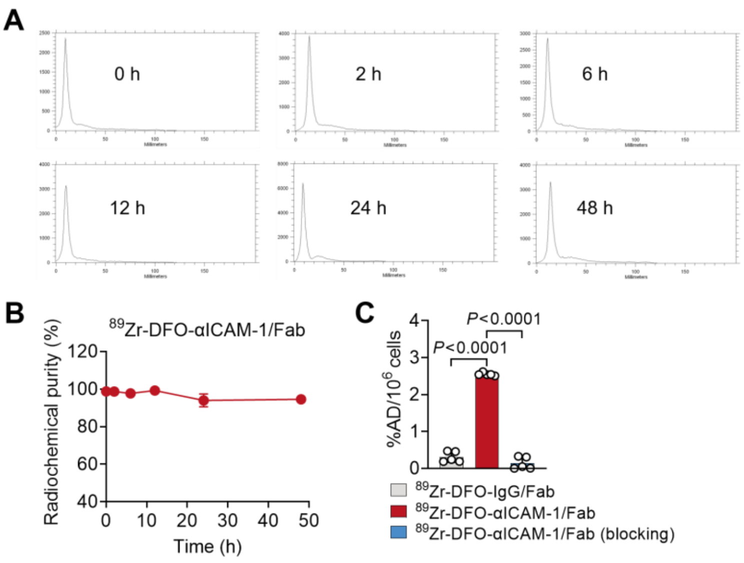

Figure S3. In vitro characterization of ^{89}Zr-DFO-αICAM-1/Fab. **A**, **B**, Representative instant thin-layer chromatography (**A**) and corresponding quantitative analysis of radiochemical purity (**B**) of ^{89}Zr-DFO-αICAM-1/Fab after incubation in PBS for 0, 2, 6, 12, 24, and 48 h (n = 3). **C**, Cell binding assay comparing ^{89}Zr-DFO-IgG/Fab and ^{89}Zr-DFO-αICAM-1/Fab with or without blocking using excess DFO-αICAM-1/Fab (n = 5 per group). Data are presented as mean ± SD. *P* values were determined by one-way ANOVA with a post hoc Tukey's test (**C**).

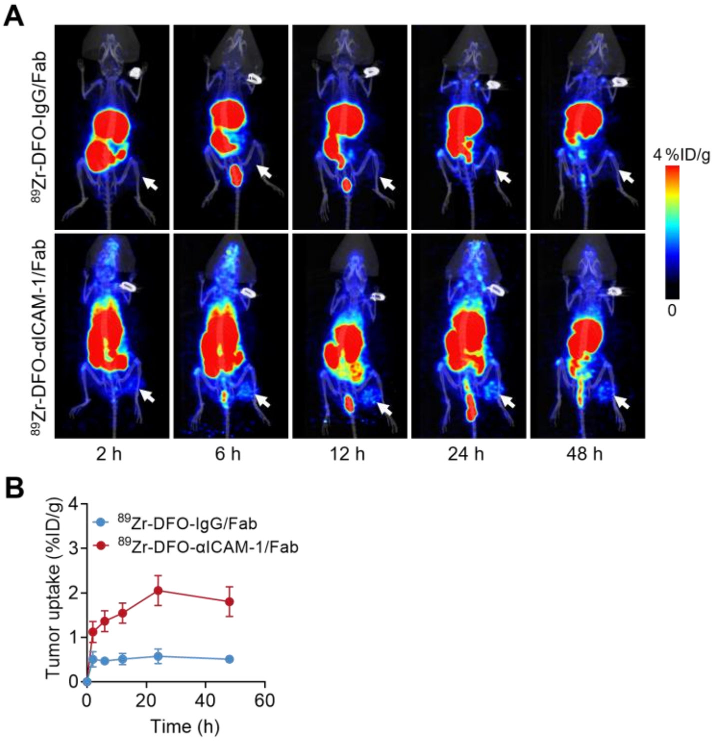

Figure S4. In vivo ICAM-1-targeting specificity of ^{89}Zr-DFO-αICAM-1/Fab. **A**, Representative PET/CT images of mice injected with ^{89}Zr-DFO-αICAM-1/Fab or ^{89}Zr-DFO-IgG/Fab at 2, 6, 12, 24, 48 h post-injection. Tumors are indicated by white arrows. **B**, Quantitative analysis of tumor uptake of ^{89}Zr-DFO-αICAM-1/Fab and ^{89}Zr-DFO-IgG/Fab. Data are presented as mean ± SD (n = 3–4 per group).

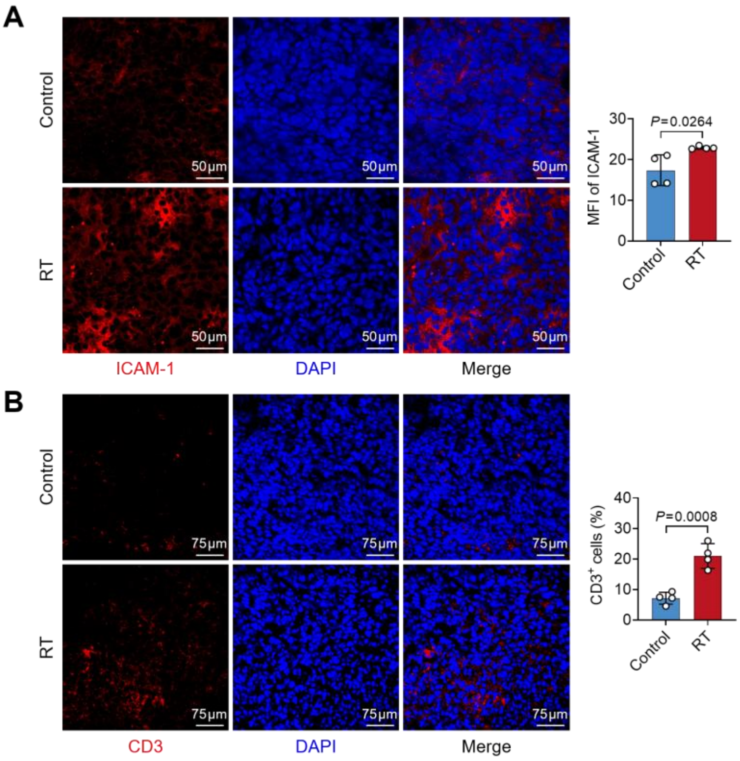

Figure S5. RT induces ICAM-1 upregulation and T cells infiltration in tumor tissues. **A**, Immunofluorescence staining and quantification of ICAM-1 expression in MC38 tumor tissues (n = 4 per group). **B**, Immunofluorescence staining and quantification of $CD3^{+}$ T cells in MC38 tumor tissues (n = 4 per group). Data are presented as mean ± SD. *P* values were determined by an unpaired Student's *t* test (**A**, **B**).

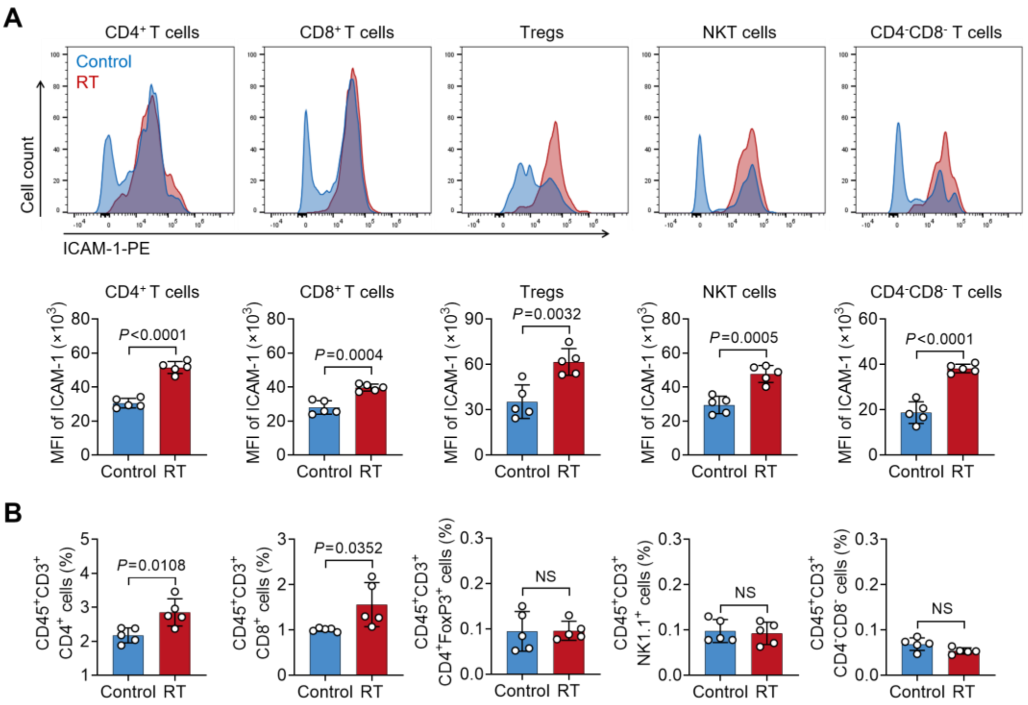

Figure S6. RT induces ICAM-1 upregulation predominantly in tumor-infiltrating CD4^{+} and CD8^{+} T-cell subsets. **A**, Representative flow cytometric histograms and quantification of ICAM-1 expression on CD4^{+} T cells, CD8^{+} T cells, regulatory T cells (Tregs), natural killer T cells (NKT cells), and CD4^{-}CD8^{-} T cells in MC38 tumors (n = 5 per group). **B**, Frequencies of CD4^{+} T cells (CD45^{+}CD3^{+}CD4^{+} cells), CD8^{+} T cells (CD45^{+}CD3^{+}CD8^{+} cells), Tregs (CD45^{+}CD3^{+}CD4^{+}FoxP3^{+} cells), NKT cells (CD45^{+}CD3^{+}NK1.1^{+} cells), and CD4^{-}CD8^{-} T cells (CD45^{+}CD3^{+}CD4^{-}CD8^{-} cells) in MC38 tumors (n = 5 per group). All numerical data are presented as mean ± SD. *P* values were determined by an unpaired Student's *t* test (**A**, **B**). NS, not significant ($P > 0.05$).

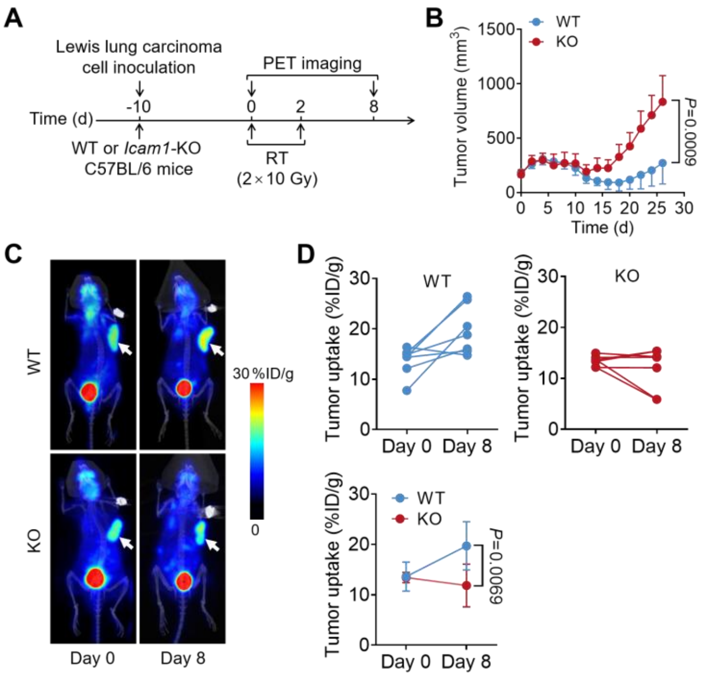

Figure S7. Genetic ablation of ICAM-1 abrogates ^{18}F-FDG flares in Lewis lung carcinoma after RT. **A**, Schedule of RT and PET imaging in WT or *Icam1*-KO C57BL/6 mice bearing Lewis lung carcinoma. **B**, Tumor growth curves post-RT in the WT and *Icam1*-KO mice (n = 7 per group). **C**, **D**, Representative PET/CT images (**C**) and quantification of ^{18}F-FDG tumor uptake (**D**) on days 0 and 8 (n = 7 per group). Tumors are indicated by white arrows. All numerical data are presented as mean ± SD. *P* values were determined by two-way ANOVA (**B**) and an unpaired Student's *t* test (**D**).

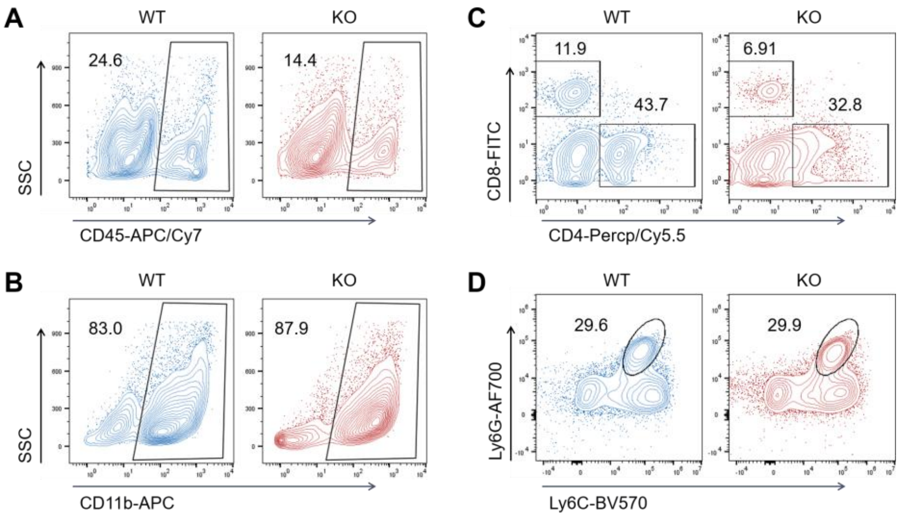

Figure S8. Flow cytometric analysis of MC38 tumors from WT or *Icam1*-KO mice post-RT. **A**–**D**, Representative flow cytometric plots showing the proportions of $CD45^+$ cells among total cells (**A**) and $CD11b^+$ (**B**), $CD4^+$ and $CD8^+$ (**C**), and $CD11b^+Ly6G^+Ly6C^-$ (**D**) cells among $CD45^+$ cells. Data related to Figure 3F in the main text.

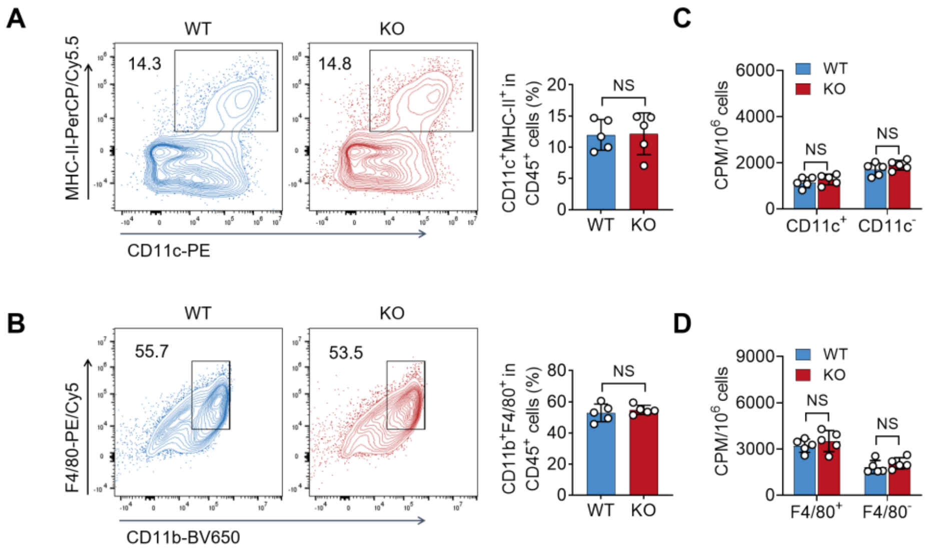

Figure S9. Genetic ablation of ICAM-1 does not affect tumor infiltration or ^{18}F-FDG uptake in dendritic cells and macrophages. **A**, **B**, Representative flow cytometric plots and quantification of CD11c$^+$MHC-II$^+$ cells (**A**) and CD11b$^+$F4/80$^+$ cells (**B**) among CD45$^+$ cells (n = 5 per group). **C**, **D**, ^{18}F-FDG avidity of CD11c$^+$ and CD11c$^-$ cells (**C**) and F4/80$^+$ and F4/80$^-$ cells (**D**) isolated by magnetic-activated cell sorting from MC38 tumor tissues (n = 5 per group). All numerical data are presented as mean ± SD. *P* values were determined by an unpaired Student's *t* test (**A**–**D**). NS, not significant ($P > 0.05$).

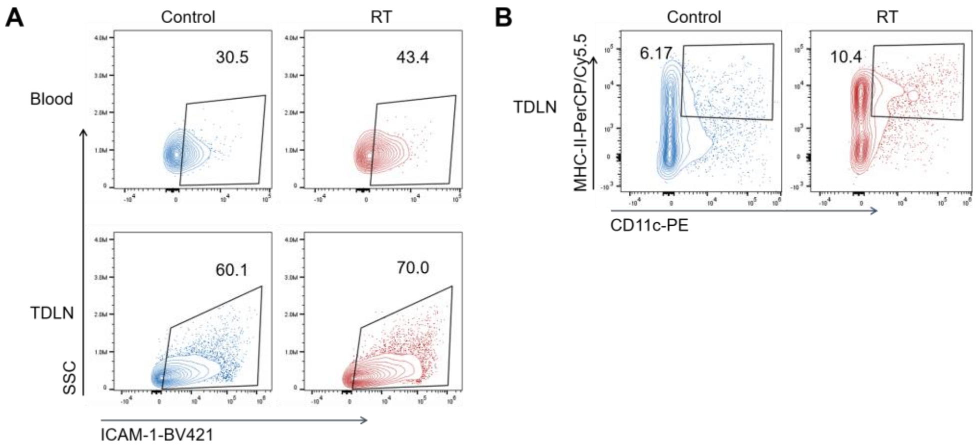

Figure S10. Flow cytometric analysis of ICAM-1$^+$ T cells and activated dendritic cells post-RT. **A**, Flow cytometric analysis showing the frequencies of ICAM-1$^+$ cells among CD45$^+$CD3$^+$ cells in blood and TDLNs. **B**, Frequencies of CD11c$^+$MHC-II$^+$ cells among CD45$^+$ cells in TDLNs. Data related to Figure 4B in the main text.

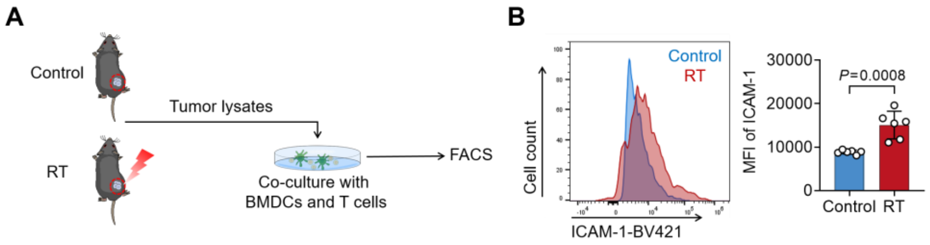

Figure S11. RT-induced ICAM-1 upregulation on T cells co-cultured with BMDCs. **A**, Schematic illustration of the experimental design: T cells were co-cultured with BMDCs stimulated by protein lysates obtained from MC38 tumor tissues harvested from mice with or without RT treatment. **B**, Representative flow cytometric histogram and quantification of ICAM-1 expression on T cells after co-culturing with BMDCs (n = 6 per group). Data are presented as mean ± SD. *P* value was determined by an unpaired Student's *t* test (**B**).

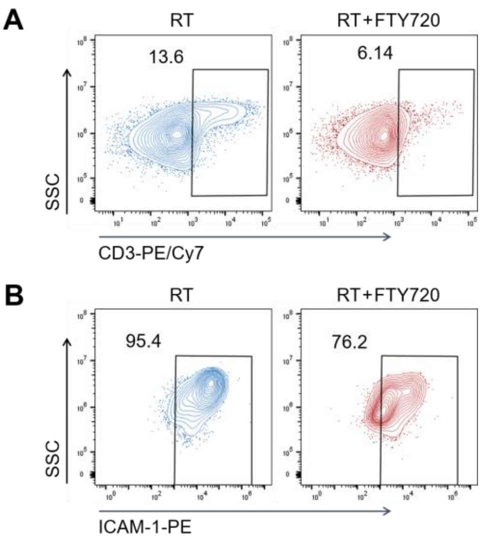

Figure S12. FTY720 treatment reduces CD3^{+} and ICAM-1^{+} T cells in irradiated tumors. **A**, **B**, Representative flow cytometric plots showing frequencies of CD3^{+} cells among CD45^{+} cells (**A**) and ICAM-1^{+} cells among CD45^{+}CD3^{+} cells (**B**) in tumors from MC38 tumor-bearing mice receiving indicated treatments. Data related to Figure 4D in the main text.

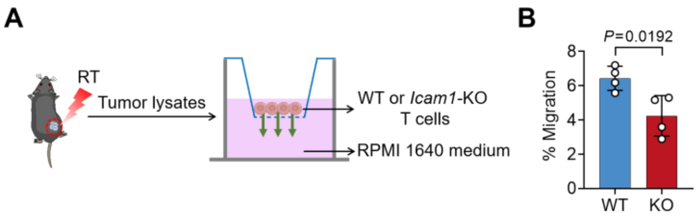

Figure S13. ICAM-1 deficiency impairs T cell migration. **A**, Schematic illustration of the transwell migration assay comparing WT and *Icam1*-KO T cells. **B**, Quantification of migrated T cells in the lower chamber, expressed as a percentage of total input cells (n = 4 per group). Data are presented as mean ± SD. *P* value was determined by an unpaired Student's *t* test (**B**).

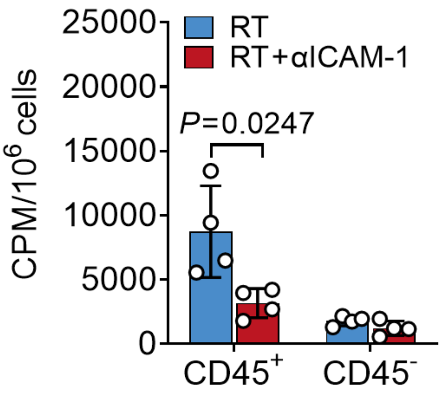

Figure S14. ICAM-1 blockade reduces ^{18}F-FDG uptake in $CD45^+$ tumor-infiltrating cells post-RT. ^{18}F-FDG avidity of $CD45^+$ and $CD45^-$ cells isolated by magnetic-activated cell sorting from tumors treated with RT or RT plus anti-ICAM-1 antibody (RT + αICAM-1) on day 8 (n = 4 per group). Data are presented as mean ± SD. *P* value was determined by an unpaired Student's *t* test.

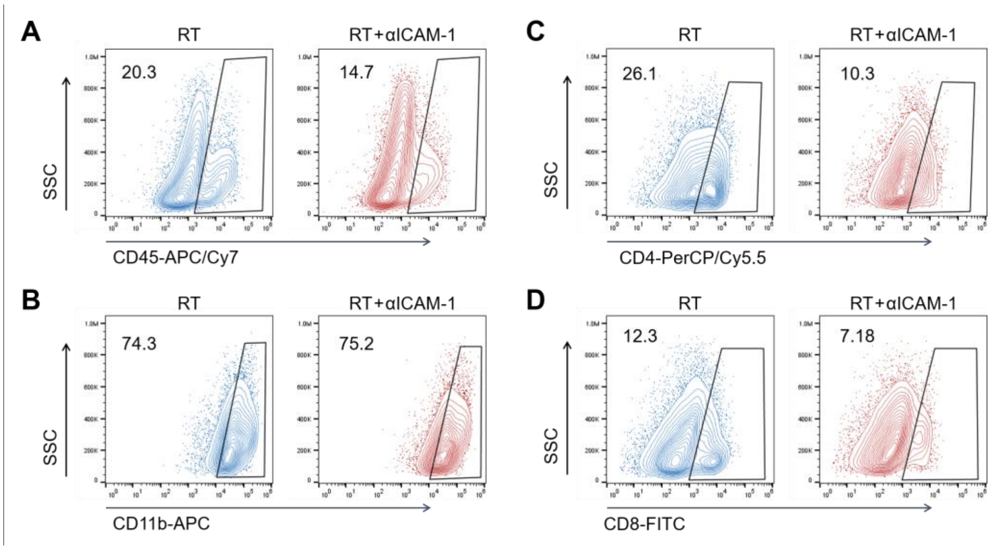

Figure S15. Flow cytometric profiling of MC38 tumors treated with RT or RT plus anti-ICAM-1 antibody (RT + αICAM-1). **A**–**D**, Representative flow cytometric plots showing frequencies of $CD45^+$ cells among total cells (**A**) and $CD11b^+$ (**B**), $CD4^+$ (**C**), and $CD8^+$ (**D**) cells among $CD45^+$ cells in tumor tissues. Data related to Figure 4I in the main text.

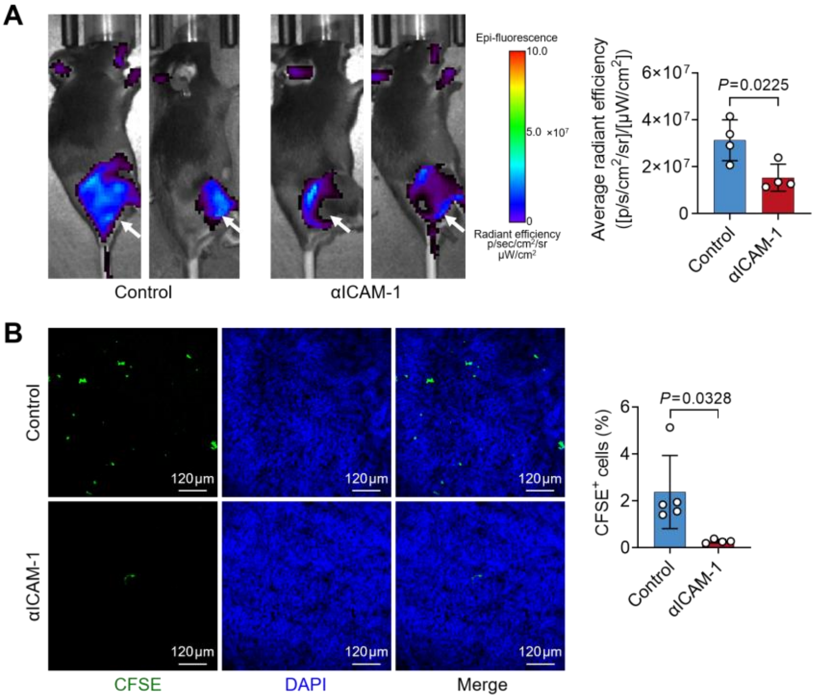

Figure S16. ICAM-1 blockade reduces T cell infiltration into tumors. **A**, Representative near-infrared fluorescence images and corresponding quantification of DiR-labeled T cells in MC38 tumor-bearing mice 24 h after adoptive transfer, with or without anti-ICAM-1 antibody (αICAM-1) pre-treatment (n = 4 per group). Tumors are indicated by white arrows. **B**, Immunofluorescence staining and quantification of CFSE-labeled T cells in MC38 tumors 24 h after adoptive transfer, with or without αICAM-1 pre-treatment (n = 4–5 per group). Data are presented as mean ± SD. *P* values were determined by an unpaired Student's *t* test (**A**, **B**).

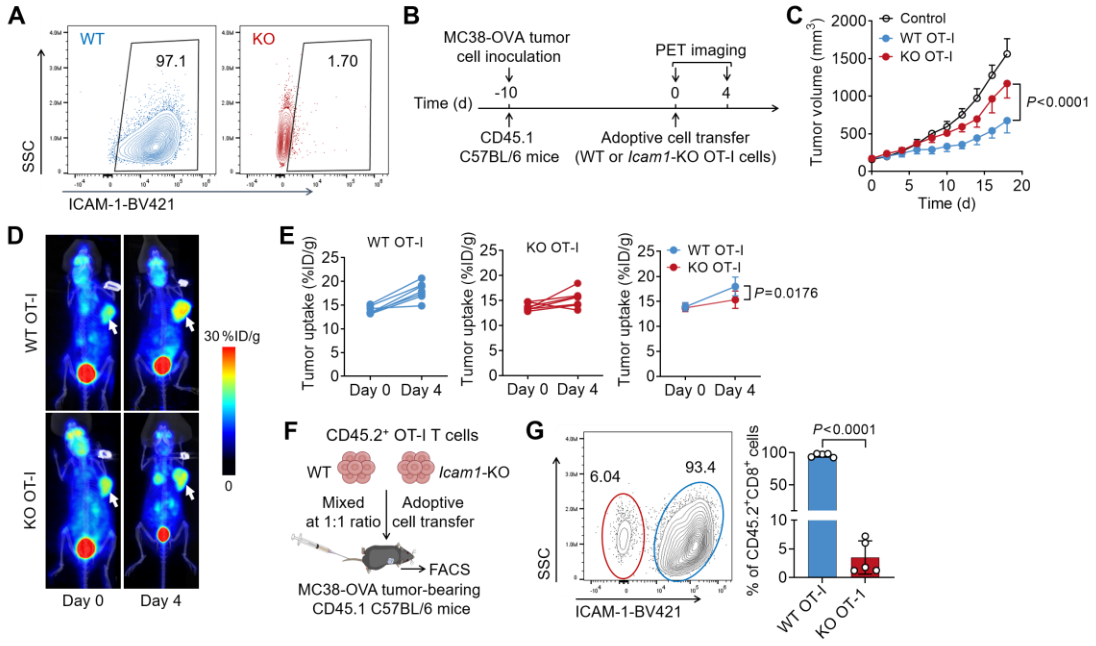

Figure S17. T cell-intrinsic ICAM-1 promotes tumor infiltration capacity of OT-I T cells. **A**, Flow cytometric analysis of ICAM-1 expression on WT and *Icam1*-KO OT-I T cells. **B**, Schedule of adoptive transfer of WT or *Icam1*-KO OT-I T cells into MC38-OVA tumor-bearing CD45.1^{+} C57BL/6 mice. **C**, Tumor growth curves of CD45.1^{+} C57BL/6 mice bearing MC38-OVA tumors following the indicated treatments: PBS (control), adoptive transfer of WT OT-I T cells (WT OT-I), or adoptive transfer of *Icam1*-KO OT-I T cells (KO OT-I) (n = 7 per group). **D**, **E**, Representative PET/CT images (**D**) and quantification of ^{18}F-FDG tumor uptake (**E**) on days 0 and 4 after adoptive transfer of WT OT-I or *Icam1*-KO OT-I T cells (n = 7 per group). Tumors are indicated by white arrows. **F**, Experimental scheme of co-transfer of a 1:1 mixture of WT and *Icam1*-KO CD45.2^{+} OT-I T cells into MC38-OVA tumor-bearing CD45.1^{+} C57BL/6 mice (n = 5). **G**, Representative flow cytometric plots and quantification of WT and *Icam1*-KO cells among CD45.2^{+}CD8^{+} tumor-infiltrating cells. All numerical data are presented as mean ± SD. *P* values were determined by two-way ANOVA (**C**) and an unpaired Student's *t* test (**E**, **G**).

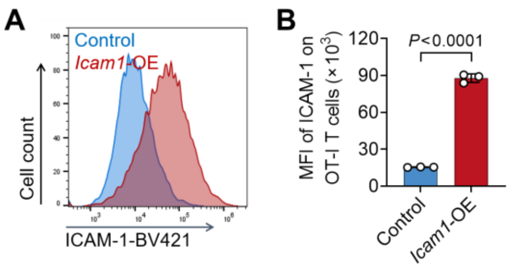

Figure S18. Characterization of *Icam1*-OE OT-I T cells. **A**, **B**, Representative flow cytometric histogram (**A**) and quantification (**B**) of ICAM-1 expression in WT OT-I (control) and *Icam1*-OE (*Icam1*-OE) OT-I T cells. Data are presented as mean ± SD (n = 3 per group). *P* value was determined by an unpaired Student's *t* test (**B**).

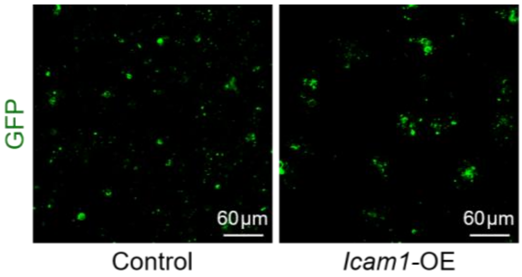

Figure S19. ICAM-1 overexpression promotes T cell cluster formation. Formation of T cell clusters in WT OT-I (control) and *Icam1*-OE OT-I T cells transfected with green fluorescent protein (GFP).

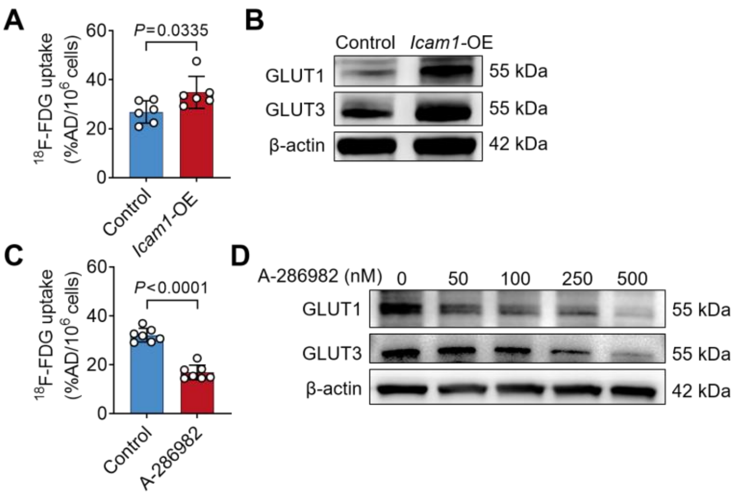

Figure S20. ICAM-1 enhances ^{18}F-FDG uptake in T cells via interaction with LFA-1. **A**, **B**, In vitro ^{18}F-FDG uptake (**A**) and western blot analysis of GLUT1 and GLUT3 (**B**) in WT (control) and *Icam1*-OE T cells. **C**, **D**, In vitro ^{18}F-FDG uptake (**C**) and western blot analysis of GLUT1 and GLUT3 (**D**) in T cells with or without A-286982 pre-treatment. Data are presented as mean ± SD (n = 6–7 per group). *P* values were determined by an unpaired Student's *t* test (**A**, **C**).

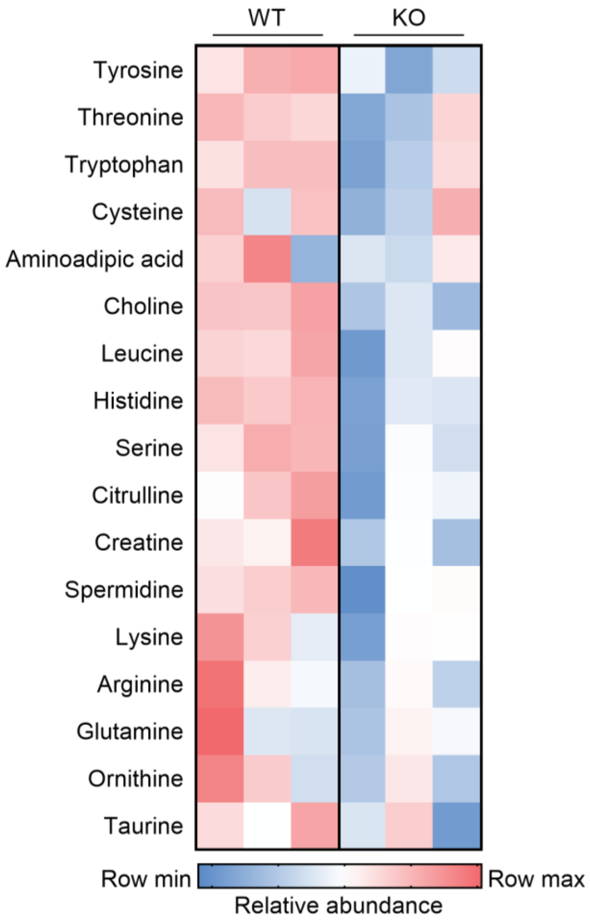

Figure S21. Heatmap of relative amino acid concentrations per row in WT and *Icam1*-KO T cells (n = 3 per group). Red and blue indicate high and low abundances, respectively.

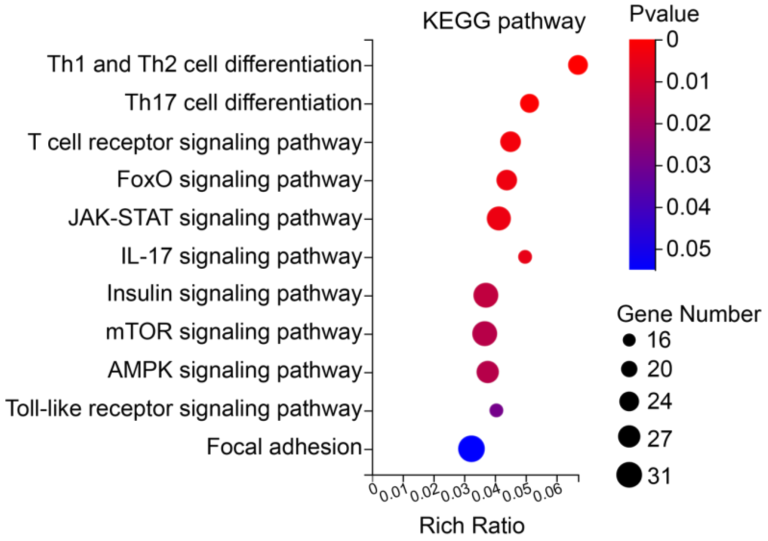

Figure S22. Kyoto Encyclopedia of Genes and Genomes (KEGG) pathway analysis of WT and *Icam1*-KO T cells. RNA-seq data showing representative downregulated signaling pathways in *Icam1*-KO versus WT T cells (n = 3 per group).

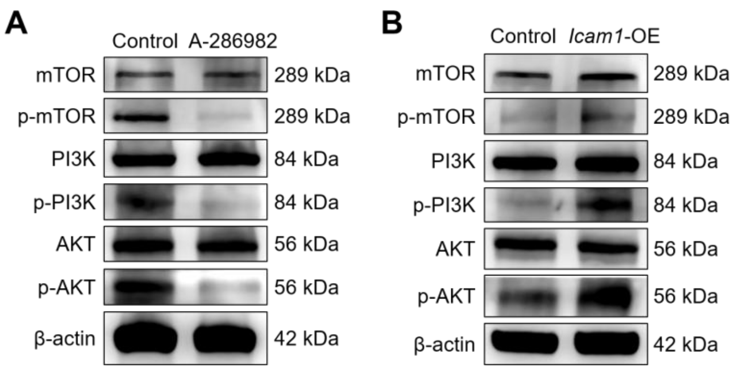

Figure S23. ICAM-1 mediates the PI3K-AKT-mTOR signaling pathway through interaction with LFA-1 on T cells. **A**, Western blot analysis of non-phosphorylated and phosphorylated mTOR, PI3K, and AKT in T cells with or without pre-treatment with A-286982. **B**, Western blot analysis of non-phosphorylated and phosphorylated mTOR, PI3K, and AKT in control and *Icam1*-OE T cells.

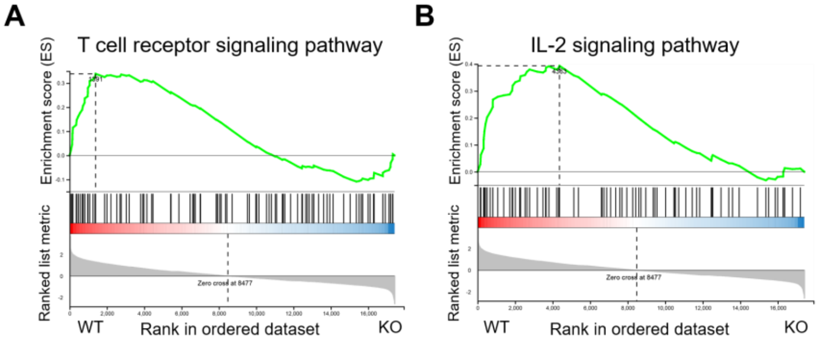

Figure S24. Genetic ablation of ICAM-1 suppresses T cell effector function. **A**, **B**, GSEA of T cell receptor (**A**) and IL-2 signaling pathways (**B**) in WT versus *Icam1*-KO T cells (n = 3 per group).

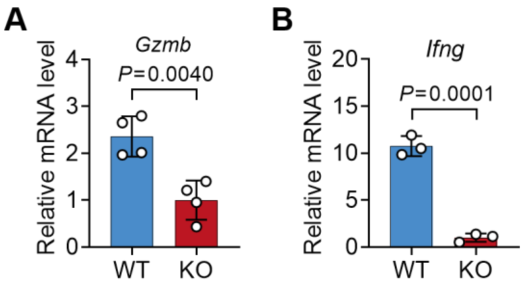

Figure S25. Genetic ablation of ICAM-1 suppresses T cell effector function at the mRNA level. **A**, **B**, Quantitative real-time PCR analysis of *Gzmb* (**A**) and *Ifng* (**B**) expression in WT and *Icam1*-KO T cells. Data are presented as mean ± SD (n = 3–4 per group). *P* values were determined by an unpaired Student's *t* test (**A**, **B**).

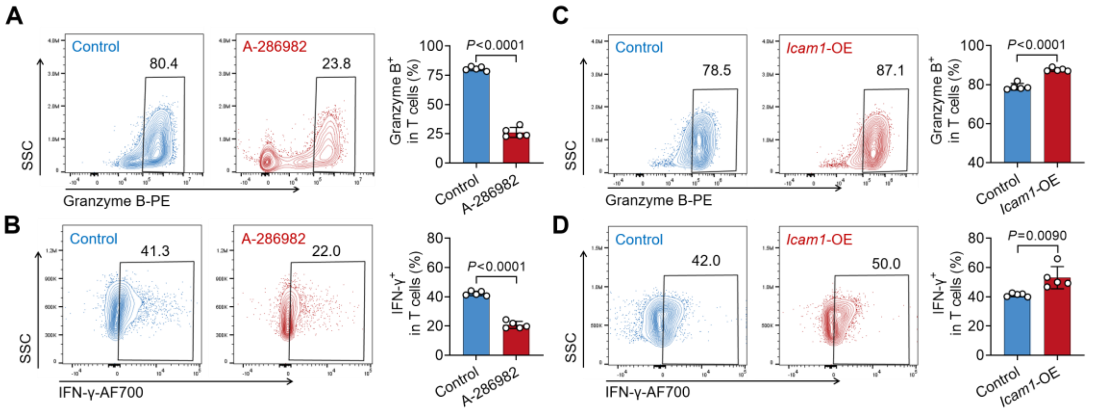

Figure S26. ICAM-1 enhances the effector function of T cells through interaction with LFA-1. **A**, **B**, Representative flow cytometric plots and quantification of granzyme B$^+$ (**A**) and IFN-γ$^+$ (**B**) cells in T cells with or without pre-treatment with A-286982 (n = 5 per group). **C**, **D**, Representative flow cytometric plots and quantification of granzyme B$^+$ (**C**) and IFN-γ$^+$ (**D**) cells in WT (control) and *Icam1*-OE T cells (n = 5 per group). All numerical data are presented as mean ± SD. *P* values were determined by an unpaired Student's *t* test (**A**–**D**).

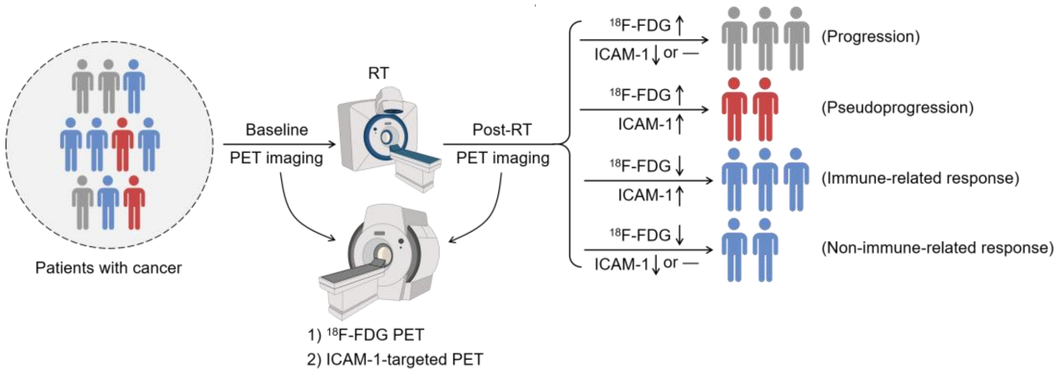

Figure S27. Proposed clinical imaging strategies combining ^{18}F-FDG PET and ICAM-1-targeted PET for precise assessment of tumor responses to RT.

Table S1. Patient characteristics.

Patient #	Gender	Age (Y)	Tumor type	Stage	Therapy regimens	^{18}F-FDG PET/CT							RT date
						**Tumor size (cm)**		**SUV_{max}**		**SUL_{peak}**		**Response evaluation**	
						**Before treatment**	**After treatment**	**Before treatment**	**After treatment**	**Before treatment**	**After treatment**		
1	Female	70	Lung cancer	cT1N0M0 (I)	Stereotactic radiotherapy (95% PTV 50 Gy/5 f)	1.77 × 1.46	3.59 × 1.82	2.61	4.36	1.62	3.48	PMD (PERCIST)	02/20/2021-02/24/2021
2	Male	55	NK/T-cell lymphoma	IVB	GELOX (1 cycle), GELOX + temozolomide (1 cycle); radiotherapy (50 Gy/25 f); P-GeMox (2 cycles); CHOPE (2 cycles)	4.92 × 2.63 (baseline); 0 × 0 (before RT)	3.03 × 2.43	8.90 (baseline); NA (before RT)	4.12	5.92 (baseline); NA (before RT)	4.37	PD (Lugano)	08/28/2017-10/02/2017

Abbreviations: SUVmax, maximum standardized uptake value; SULpeak, peak standardized uptake value corrected for lean body mass; RT, radiotherapy; PTV, planning target volume; GELOX, gemcitabine + oxaliplatin + dexamethasone + peiaspartase; P-GeMox, gemcitabine + oxaliplatin + aspartase + dexamethasone; CHOPE, cyclophosphamide + vindesine + methylprednisolone + etoposide + epirubicin; NA, not available; PMD, progressive metabolic disease; PD, progressive disease.

Table S2. Radiotherapy administrations and human tumor sample collection time points.

Patient #	Tumor type	Gender	Age (Y)	RT dose	RT date	Sample collection date	
						Before RT	After RT
1	Middle-differentiated adenocarcinoma of the rectum	Male	68	95% PTV45: 45 Gy/25 f; 95% PTV50: 50 Gy/25 f; 95% PTV60: 60 Gy/25 f	12/07/2023–01/18/2024	11/06/2023	03/26/2024
2		Male	70	95% PTV45: 45 Gy/25 f; 95% PTV50: 50 Gy/25 f	02/15/2024–03/20/2024	01/25/2024	06/04/2024
3		Male	45	95% PTV45: 45 Gy/25 f; 95% PTV50: 50 Gy/25 f	03/31/2023–05/08/2023	03/01/2023	07/14/2023
4		Male	69	95% PTV45: 45 Gy/25 f; 95% PTV50: 50 Gy/25 f	03/31/2023–05/08/2023	03/10/2023	07/29/2023

Abbreviations: 95% PTV45 is applied to a 3D expansion of 5 mm (7 mm posteriorly) from areas including obturator, internal iliac, presacral lymphatic drainage, and the rectosigmoid mesentery; 95% PTV50 is applied to a 3D expansion of 5 mm beyond the rectosigmoid mesentery area; 95% PTV60 is applied to a 3D expansion of 5 mm beyond the enlarged pelvic lymph nodes.

Table S3. Fluorescently labeled antibodies used in this study for flow cytometric analysis.

Reagent	Source	Identifier
Anti-mouse CD45 (APC)	BioLegend	Clone: 30-F11; Cat#: 103112
Anti-mouse CD45 (APC/Cy7)	BioLegend	Clone: 30-F11; Cat#: 103116
Anti-mouse CD45.2 (APC)	BioLegend	Clone: 104; Cat#: 109814
Anti-mouse CD3 (BV750)	BioLegend	Clone: 17A2; Cat#: 100249
Anti-mouse CD4 (PerCP/Cy5.5)	eBioscience	Clone: RM4-5; Cat#: 45-0042-80
Anti-mouse FoxP3 (PE/Cy7)	eBioscience	Clone: FJK-16s; Cat#: 25-5773-82
Anti-mouse CD8 (FITC)	eBioscience	Clone: 53-6.7; Cat#: 11-0081-82
Anti-mouse CD8 (PE)	BioLegend	Clone: 53-6.7; Cat#: 100707
Anti-mouse CD11b (APC)	BioLegend	Clone: M1/70; Cat#: 101212
Anti-mouse CD11b (BV650)	eBioscience	Clone: M1/70; Cat#: 416-0112-80
Anti-mouse F4/80 (PE/Cy5)	BioLegend	Clone: BM8; Cat#: 123111
Anti-mouse Ly6C (BV570)	BioLegend	Clone: HK1.4; Cat#: 128029
Anti-mouse Ly6G (AF700)	BioLegend	Clone: 1A8; Cat#: 127621
Anti-mouse CD11c (PE)	BioLegend	Clone: N418; Cat#: 117307
Anti-mouse MHC-II (PerCP/Cy5.5)	BioLegend	Clone: M5/114.15.2; Cat#: 107625
Anti-mouse NK1.1 (APC/Cy7)	BioLegend	Clone: S17016D; Cat#: 156509
Anti-mouse CD19 (PE/Cy7)	BioLegend	Clone: 1D3/CD19; Cat#: 152417
Anti-mouse CD31 (FITC)	BioLegend	Clone: 390; Cat#: 102405
Anti-mouse ICAM-1 (PE)	eBioscience	Clone: YN1/1.7.4; Cat#: 12-0541-81
Anti-mouse ICAM-1 (BV421)	BioLegend	Clone: YN1/1.7.4; Cat#: 116141
Anti-mouse granzyme B (PE)	BioLegend	Clone: QA16A02; Cat#: 372208
Anti-mouse IFN-γ (AF700)	BioLegend	Clone: XMG1.2; Cat#: 505824
